# Targeted H_2_S Delivery System Attenuates Blood‐Spinal Cord Barrier Disruption after Spinal Cord Injury by Reshaping the Ferritinophagy Pathway

**DOI:** 10.1002/advs.202518901

**Published:** 2026-03-10

**Authors:** Zhiheng Chen, Xinkai Pu, Ruiyang Li, Yuezhou Wu, Qirong Zhou, Jian Wang, Yuxuan Qian, Fengjie Lu, Jian‐Yuan Zhao, Zhida Su, Jiacan Su, Xiaofeng Lian

**Affiliations:** ^1^ Department of Orthopedics Shanghai Sixth People's Hospital Affiliated to Shanghai Jiao Tong University School of Medicine Shanghai China; ^2^ Department of Orthopedics Xinhua Hospital Affiliated to Shanghai Jiao Tong University School of Medicine Shanghai China; ^3^ Institute of Translational Medicine Shanghai University Shanghai China; ^4^ MedEng‐X Institutes Shanghai University Shanghai China; ^5^ State Key Laboratory of Systems Medicine For Cancer, Department of Urology, Shanghai Cancer Institute, Ren Ji Hospital School of Medicine, Shanghai Jiao Tong University Shanghai China; ^6^ Ministry of Education‐Shanghai Key Laboratory of Children's Environmental Health, Institute for Developmental and Regenerative Cardiovascular Medicine, Xinhua Hospital Shanghai Jiao Tong University School of Medicine Shanghai China; ^7^ Institute of Neuroscience and Key Laboratory of Molecular Neurobiology of Ministry of Education Naval Medical University Shanghai China

**Keywords:** blood spinal cord barrier, ferritinophagy, H_2_S, spinal cord injury, targeted nanoparticles

## Abstract

As a severe and disabling central nervous system disorder, spinal cord injury (SCI) remains challenging, partly because of the difficulty in addressing secondary injury caused by the blood‐spinal cord barrier (BSCB) disruption. As the neurovascular unit's crucial component, the BSCB regulates the homeostasis of the spinal cord. Inspired by the established protective effect of H_2_S in pan‐vascular pathologies, we engineered an intravenously administered nanoparticle SPRC@MPDA‐RGD. By utilizing the overexpression of α_v_β_3_ integrin on endothelial cells after SCI, the functionalized peptide c(RGDyK) can guide SPRC@MPDA‐RGD for precise delivery to the BSCB. The MPDA scaffold has the ability to both deliver S‐propargyl‐cysteine (SPRC) and scavenge reactive oxygen species (ROS). Subsequently, the release of SPRC upregulates cystathionine γ‐lyase (CSE) and stimulates endogenous H_2_S production in injured endothelial cells, thereby protecting the BSCB. We also investigated the biological mechanisms underlying the therapeutic effects of SPRC@MPDA‐RGD. The production of H_2_S in endothelial cells activates the PI3K/Akt/mTOR pathway, which subsequently suppresses ferritinophagy, reduces ferritin degradation, and ultimately suppresses ferroptosis. In summary, our work proposes a nanotherapeutic strategy that coordinates H_2_S production and ROS scavenging to inhibit ferritinophagy, thereby promoting BSCB repair, showing significant potential in promoting SCI treatment.

## Graphical abstract

The α_v_β_3_‐mediated SPRC@MPDA‐RGD targets broken endothelial cells and controllably releases SPRC. CSE is then activated to produce endogenous H_2_S, which inhibits ferritinophagy. In brief, H_2_S inhibits autophagy by activating the PI3K/Akt/mTOR pathway, thereby suppressing the ferroptosis process mediated by NCOA4, and ultimately promoting the reconstruction of BSCB.

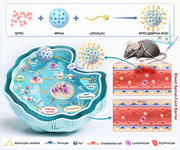

## 1 Introduction

Spinal cord injury (SCI) is a disability‐causing disease mainly caused by trauma, which brings a huge physical, mental, and socio‐economic burden to patients themselves and the whole society [1, 2], with more than 700 000 new cases reported every year [3]. However, the clinical treatment of SCI is still very limited, and the prognosis of patients is still unsatisfactory [4, 5, 6]. Pathologically, the progression of SCI has two stages: primary and secondary injury [7]. The initial mechanical insult causes immediate neuronal destruction and blood‐spinal cord barrier (BSCB) disruption, which can result in inflammatory factors, cytokines, and vasoactive peptides infiltrated into the lesion site [8, 9, 10]. This will cause secondary injuries, which will lead to the gradual aggravation of spinal cord edema and aggravate the defect of nerve function [7, 11]. Consequently, strategies targeting the early restoration of BSCB integrity represent a pivotal therapeutic avenue to limit secondary damage and improve neurological outcomes.

Endothelial cells are one of the main constituent cells of the BSCB, which can directly regulate the integrity of the barrier [12]. Hydrogen sulfide (H_2_S), a recognized gaseous mediator, demonstrates potent endothelial protective effects in cardiovascular contexts [13]. Xu et al. showed that the exogenous H_2_S donor NaHS preserves the blood‐brain barrier (BBB) integrity via suppressing autophagy and restoring mitochondrial function [14]. The research by Wang et al. demonstrated H_2_S given by NaHS exerts a protective effect on BSCB through inhibition of endoplasmic reticulum stress‐dependent autophagy post‐SCI [15]. Our study further indicated that SCI disrupts endothelial iron metabolism, causing free iron overload that triggers Fenton reactions and ferroptosis [16]. Miao et al. also indicated that H_2_S can improve endothelial dysfunction by inhibiting ferroptosis in endothelial cells [17]. Collectively, these studies emphasize the important role of H_2_S in endothelial regulation.

There are still many challenges in transforming H_2_S into the treatment of SCI. Traditional inorganic donors (e.g., NaHS, Na_2_S) have rapid and uncontrollable release kinetics, which will lead to short‐term cytotoxic H_2_S peaks, thus aggravating damage [18, 19, 20, 21]. S‐propargyl‐cysteine (SPRC), an endogenous H_2_S donor, offers a promising alternative by upregulating cystathionine γ‐lyase (CSE) expression to promote physiological H_2_S generation [22, 23]. SPRC demonstrates therapeutic efficacy across multiple disease models, promoting microvascular reconstruction in nerve injury, enhancing angiogenesis via STAT3 phosphorylation in ischemia, exerting anti‐inflammatory/antioxidant effects in arthritis, and suppressing neuroinflammation through TNF‐α/TNFR1 inhibition [24, 25, 26, 27]. Nevertheless, its clinical translation is hindered by rapid systemic clearance and poor tissue targeting specificity [28, 29]. Thus, achieving sustained and spatially controlled H_2_S delivery remains a critical challenge.

During secondary injury, infiltrated microglia/macrophages exacerbate the destruction of the BSCB by enhancing the inflammatory response, thus exacerbating neuronal apoptosis and axonal demyelination [30, 31]. Mesoporous polydopamine nanoparticles (MPDA) present an ideal delivery platform due to their biocompatibility, biodegradability, and potent ROS‐scavenging capacity via abundant phenolic groups [32, 33]. Emerging evidence has also demonstrated that MPDA has the capacity to inhibit ferroptosis through the chelation of Fe ions [34, 35]. Furthermore, MPDA features tunable mesopores that enhance drug loading capacity while enabling sustained release kinetics‐overcoming the drug leakage limitations of non‐porous polydopamine [32, 36, 37, 38, 39]. Critically, the post‐SCI upregulation of integrin α_v_β_3_ on neovascular endothelial cells provides a target for the cyclic peptide c(RGDyK), which is known for its high affinity and specificity toward this receptor [40, 41, 42]. Recent studies have confirmed that RGD‐modified biomaterials can be targeted and delivered to damaged BSCB endothelial cells [43, 44].

**SCHEME 1:**
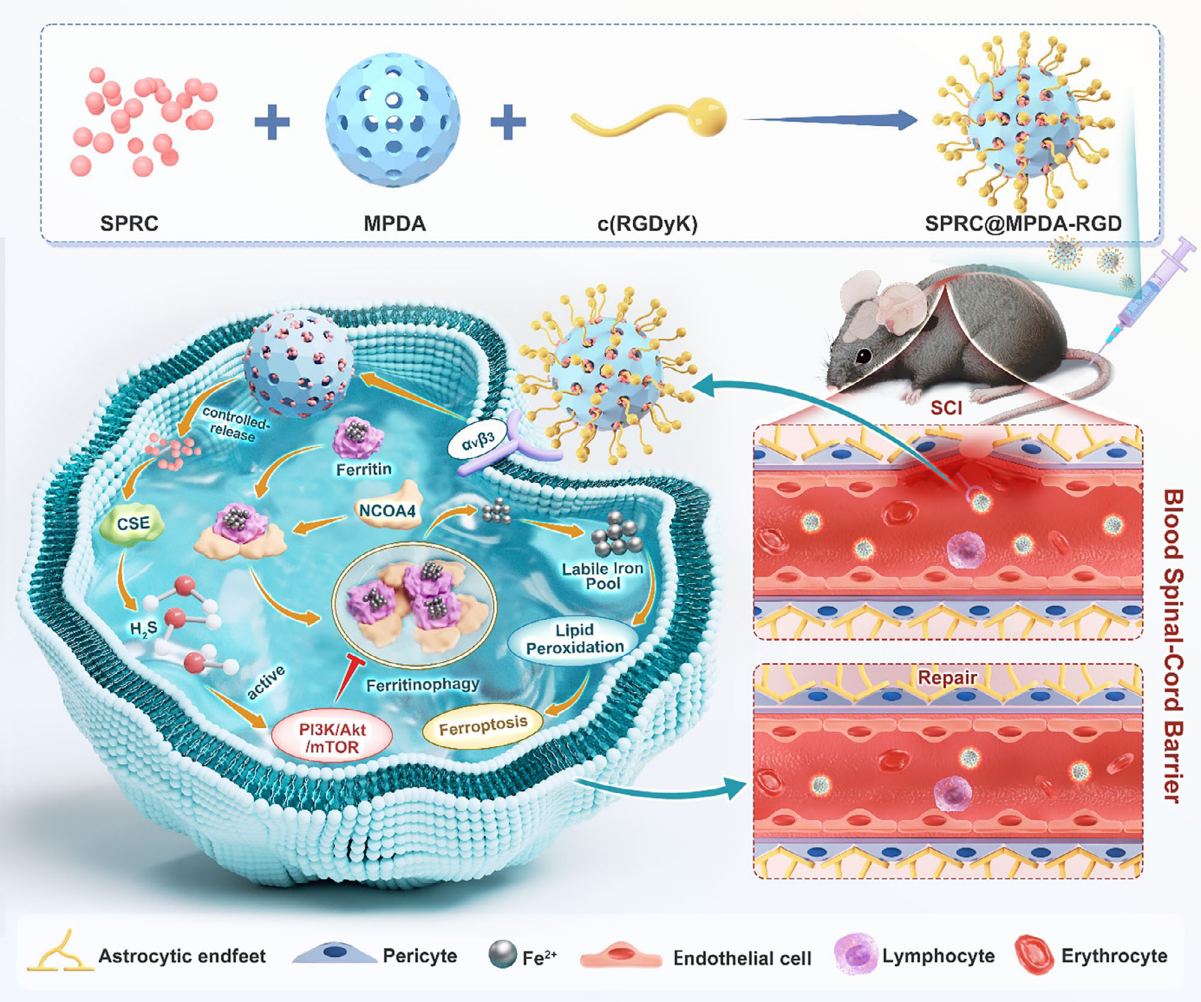
Schematic representation of SPRC@MPDA‐RGD controllably releases H_2_S for BSCB repair in SCI. The α_v_β_3_‐mediated SPRC@MPDA‐RGD targets broken endothelial cells and controllably releases SPRC. CSE is then activated to produce endogenous H_2_S, which inhibits ferritinophagy. In brief, H_2_S suppresses autophagy via activating the PI3K/Akt/mTOR signaling axis, thereby suppressing the ferroptosis process mediated by NCOA4, and ultimately promoting the reconstruction of BSCB.

In order to achieve precise delivery of BSCB endothelial cells, we designed c(RGDyK) functionalized MPDA nanoparticles (SPRC@MPDA‐RGD) to accurately repair the BSCB. The system uses c(RGDyK) to achieve active endothelial targeting, and uses MPDA's mesoporous structure for controllable H_2_S donor SPRC release at the same time. We also proved that SPRC@MPDA‐RGD can effectively target and accumulate in the damaged spinal cord, significantly enhance BSCB repair, reduce inflammation and oxidative stress, and improve functional recovery after SCI. In terms of mechanism, endogenous H_2_S plays its therapeutic role by activating the PI3K/Akt/mTOR, which maintains the integrity of the BSCB by inhibiting the ferritinophagy mediated by nuclear receptor coactivator 4 (NCOA4) (Scheme 1). This study presents a targeted strategy that orchestrates endogenous H_2_S signaling and ROS scavenging to repair the BSCB via ferritinophagy inhibition, offering a promising approach for SCI treatment.

## 2 Results

### 2.1 Preparation and Characterization of SPRC@MPDA‐RGD

**FIGURE 1:**
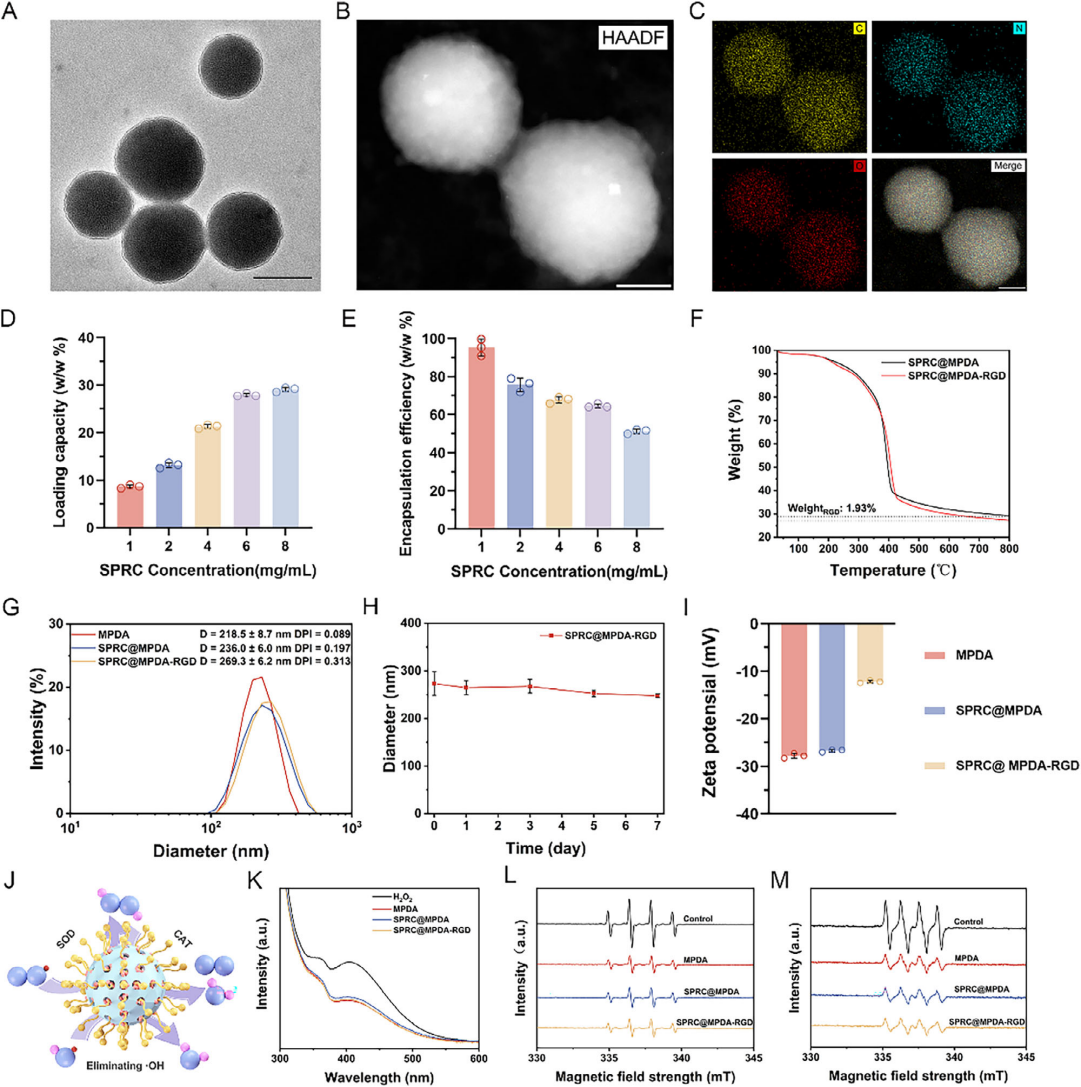
Characterizations and the multi‐enzyme mimetic properties of MPDA, SPRC@MPDA, and SPRC@MPDA‐RGD. (A) Representative TEM image. Scale bars: 200 nm. (B) HAADF image of MPDA nanoparticles. Scale bars: 100 nm. (C) The associated area‐elemental mappings of MPDA nanoparticles. Scale bars: 100 nm. (D,E) The SPRC loading capacity and encapsulation efficiency for SPRC@MPDA‐RGD (n = 3). (F) Thermogravimetric analysis results for SPRC@MPDA and SPRC@MPDA‐RGD. (G) Hydrated diameter distribution of MPDA, SPRC@MPDA, and SPRC@MPDA‐RGD measured by DLS (n = 3). (H) The hydrated diameter size of SPRC@MPDA‐RGD dispersed in PBS for 7 days (n = 3). (I) Zeta potentials of MPDA, SPRC@MPDA and SPRC@MPDA‐RGD (n = 3). (J) The schematic image of the multiple enzyme‐mimicking activities of SPRC@MPDA‐RGD. (K) Assessment of the CAT‐like activities with diverse nanoparticles using a H_2_O_2_ kit through UV–vis. (L) Electron spin resonance (ESR) curve of the scavenging hydroxyl radical (·OH) using DMPO as the spin trap. (M) ESR curve of the scavenging superoxide anion (·O_2_^−^) using DMPO as the spin trap. Data are expressed as mean ± SD.

The synthesis of MPDA nanoparticles was based on an optimized templating method [36]. And the morphology was detected through transmission electron microscopy (TEM). And MPDA presented uniform mesoporous structure, averaging about 220 nm in size (Figure 1A). In addition, high‐angle annular dark field (HAADF) further verified structural homogeneity (Figure 1B), while elemental mapping elucidated that the C, O, and N in MPDA nanoparticles were distributed uniformly (Figure 1C). SPRC was effectively loaded into the MPDA mesopores via diffusion, forming SPRC@MPDA. Subsequent surface functionalization with c(RGDyK) was performed to obtain SPRC@MPDA‐RGD. The loading capacity and encapsulation efficiency of SPRC were evaluated through high‐performance liquid chromatography (HPLC). The results indicate the efficient SPRC encapsulation with a high loading efficiency of 27.93% (w/w%) when using a dose of 6 mg/mL (Figure 1D,E). As the concentration of SPRC increases, its encapsulation efficiency shows a gradual downward trend. Thus, 6 mg/mL SPRC was determined for the preparation of SPRC@MPDA‐RGD. We then performed a thermogravimetric analysis on both SPRC@MPDA and SPRC@MPDA‐RGD to determine the grafting density of the c(RGDyK). The results indicated that the grafting density of the c(RGDyK) was 1.93% (Figure 1F). The hydrated diameter of MPDA was 218.5 ± 8.7 nm, while that of SPRC@MPDA and SPRC@MPDA‐RGD increased to 236.0 ± 6.0 nm and 269.3 ± 6.2 nm, respectively (Figure 1G). Meanwhile, the SPRC@MPDA‐RGD could be well‐dispersed in phosphate‐buffer saline (PBS) and maintain stable mean hydrated diameters during 7 d of incubation (Figure 1H). In addition, the zeta potentials of MPDA, SPRC@MPDA, and SPRC@MPDA‐RGD are respectively −27.8, −26.7, and −12.2 mV (Figure 1I).

SPRC@MPDA‐RGD also has multiple enzyme‐mimicking activities (Figure 1J). First, SPRC@MPDA‐RGD retained the potent ROS‐scavenging capabilities inherent to MPDA. It effectively decomposed H_2_O_2_ in a concentration‐dependent manner (Figure 1K; Figure S1). Given that increased ROS is an important feature of the inflammatory microenvironment, the multi‐enzyme mimetic properties of SPRC@MPDA‐RGD are applicable. The classical Fenton reaction involves a mixed system of Fe^2+^ and H_2_O_2_ that generates hydroxyl radicals. To form DMPO‐OH, 5,5‐dimethyl‐1‐pyrroline‐1‐oxide (DMPO) was used to trap these radicals. The resulting DMPO‐OH adduct exhibits an electron paramagnetic resonance peak of 1:2:2:1. SPRC@MPDA‐RGD drastically reduced the characteristic 1:2:2:1 DMPO‐OH adduct signal intensity, comparable to MPDA and SPRC@MPDA (Figure 1L). In addition, we utilized a mixed system of xanthine/xanthine oxidase to initiate the superoxide anion (·O_2_^−^), which can be captured by DMPO to form DMPO‐OOH, whose electronic paramagnetic resonance peaks are 1:1:1:1. SPRC@MPDA‐RGD significantly attenuated the 1:1:1:1 DMPO‐OOH adduct signal, again matching the efficacy of MPDA and SPRC@MPDA (Figure 1M). Critically, neither SPRC loading nor c(RGDyK) functionalization compromised the inherent enzyme‐mimetic activities of the MPDA core. SPRC@MPDA‐RGD effectively neutralized pathologically relevant ROS, confirming its suitability for the post‐SCI oxidative microenvironment.

### 2.2 SPRC@MPDA‐RGD Effectively Alleviated ROS Levels within Endothelial Cells and Promoted the Repair of Blood‐Spinal Cord Barrier

**FIGURE 2:**
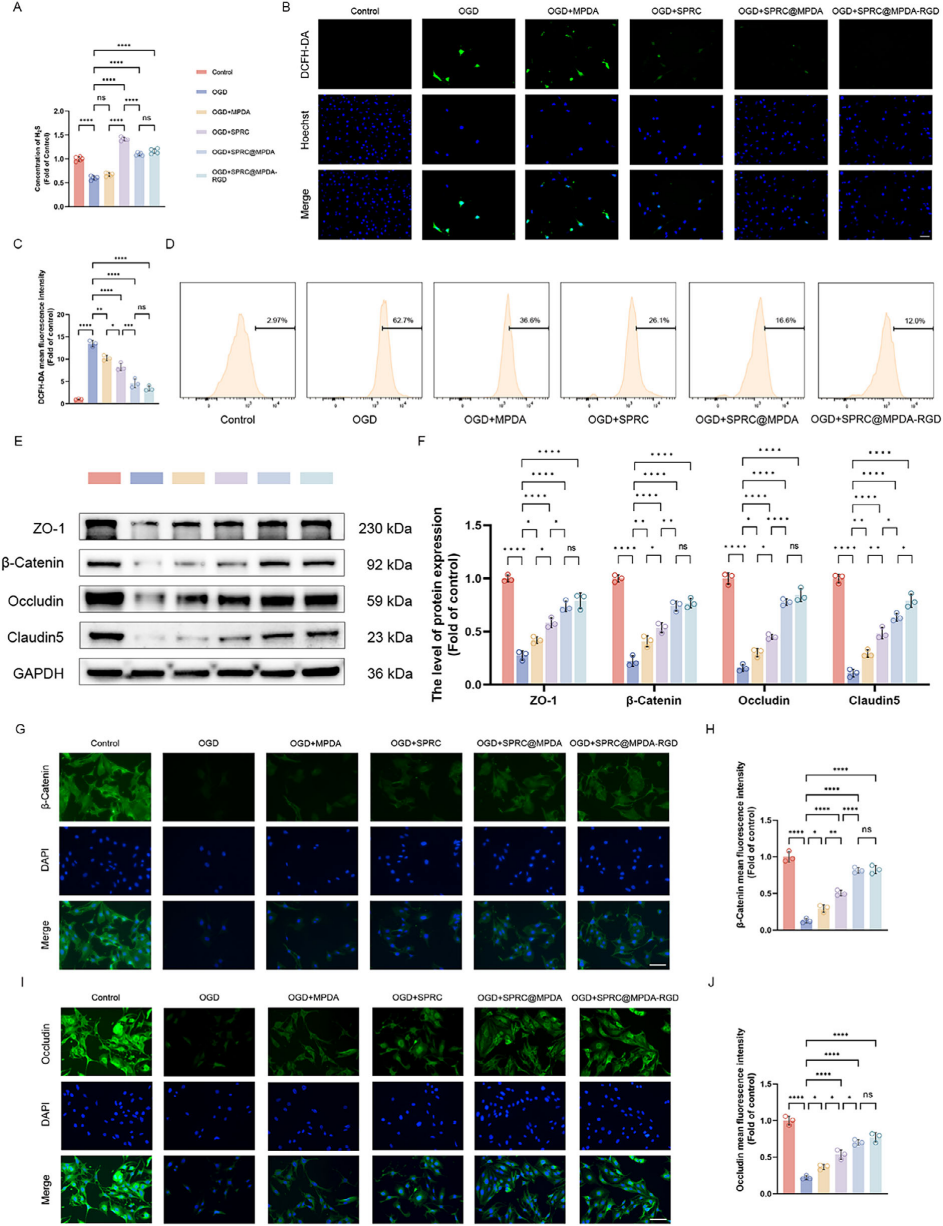
SPRC@MPDA‐RGD alleviated the oxidative stress state of endothelial cells after OGD and improved BSCB function. (A) The concentration of H_2_S in different groups measured by the endogenous H_2_S assay kit (n = 5). (B,C) Representative images of intracellular ROS levels using DCFH‐DA and the quantitative analysis results (n = 3). Scale bar = 50 µm. (D) DCFH‐DA fluorescence in bEnd.3 cells was detected and analyzed by flow cytometry. (E) The expression level of BSCB‐related protein in different groups detected by Western blotting. (F) Quantitative analysis of BSCB‐related protein expression (n = 3). (G,H) Representative immunofluorescence images of β‐Catenin and the quantitative analysis results (n = 3). Scale bar = 50 µm. (I,J) Representative immunofluorescence images of Occludin and the quantitative analysis results (n = 3). Scale bar = 50 µm. Data are expressed as mean ± SD. ^*^ *p* < 0.05, ^**^ *p* < 0.01, ^***^ *p* < 0.001, ^****^ *p* < 0.0001, ns means no significance, one‐way ANOVA with Tukey's multiple comparisons tests.

It is known that endothelial cells undergo oxidative stress after SCI, which leads to a series of functional impairments that exacerbate the destruction of the BSCB [45, 46, 47]. First, we tested the viability of bEnd.3 under the intervention of Oxygen Glucose Deprivation (OGD) through CCK‐8 assay. The results showed that 50 µg/ml SPRC@MPDA‐RGD had the greatest promoting effect (Figure S2). This dose was used for subsequent experiments. In order to verify the ability to produce H_2_S within cells, the H_2_S content in each group was measured (Figure 2A). The content of H_2_S was significantly decreased in bEnd.3 following OGD intervention. Treatment with MPDA did not significantly improve the H_2_S content, while the treatment with SPRC led to a substantial restoration of the H_2_S content in the cells. Then, we evaluated the protective effect of SPRC@MPDA‐RGD on endothelial cells under oxidative stress conditions. The results showed that MPDA exhibited antioxidant properties, and that SPRC further alleviated oxidative stress in endothelial cells. After synthesizing SPRC@MPDA‐RGD, these two components can work synergistically to reduce ROS levels in endothelial cells (Figure 2B–D). And then BSCB‐related proteins expression was determined through the western blotting. The MPDA or SPRC alone only showed moderate improvement in the decrease in BSCB‐related protein expression caused by OGD. However, SPRC@MPDA‐RGD can significantly improve the barrier damage caused by OGD (Figure 2E,F). Immunofluorescence revealed similar results. After OGD, significant decreases in β‐Catenin and Occludin were observed, while SPRC@MPDA‐RGD treatment significantly increased their expression (Figure 2G–J). The FITC‐dextran was used to assess the permeability in transwell monolayer endothelial models. OGD significantly increases the endothelial permeability, while SPRC@MPDA‐RGD effectively reduces it (Figure S3). Notably, SPRC@MPDA and SPRC@MPDA‐RGD showed no significant difference in improving the oxidative stress status and repairing BSCB‐related protein damage caused by OGD. This may suggest that in in vitro experiments, SPRC@MPDA can effectively bind to endothelial cells and exert its effects without c(RGDyK) peptides.

### 2.3 SPRC@MPDA‐RGD Targeted Vascular Endothelial Cell after Spinal Cord Injury

**FIGURE 3:**
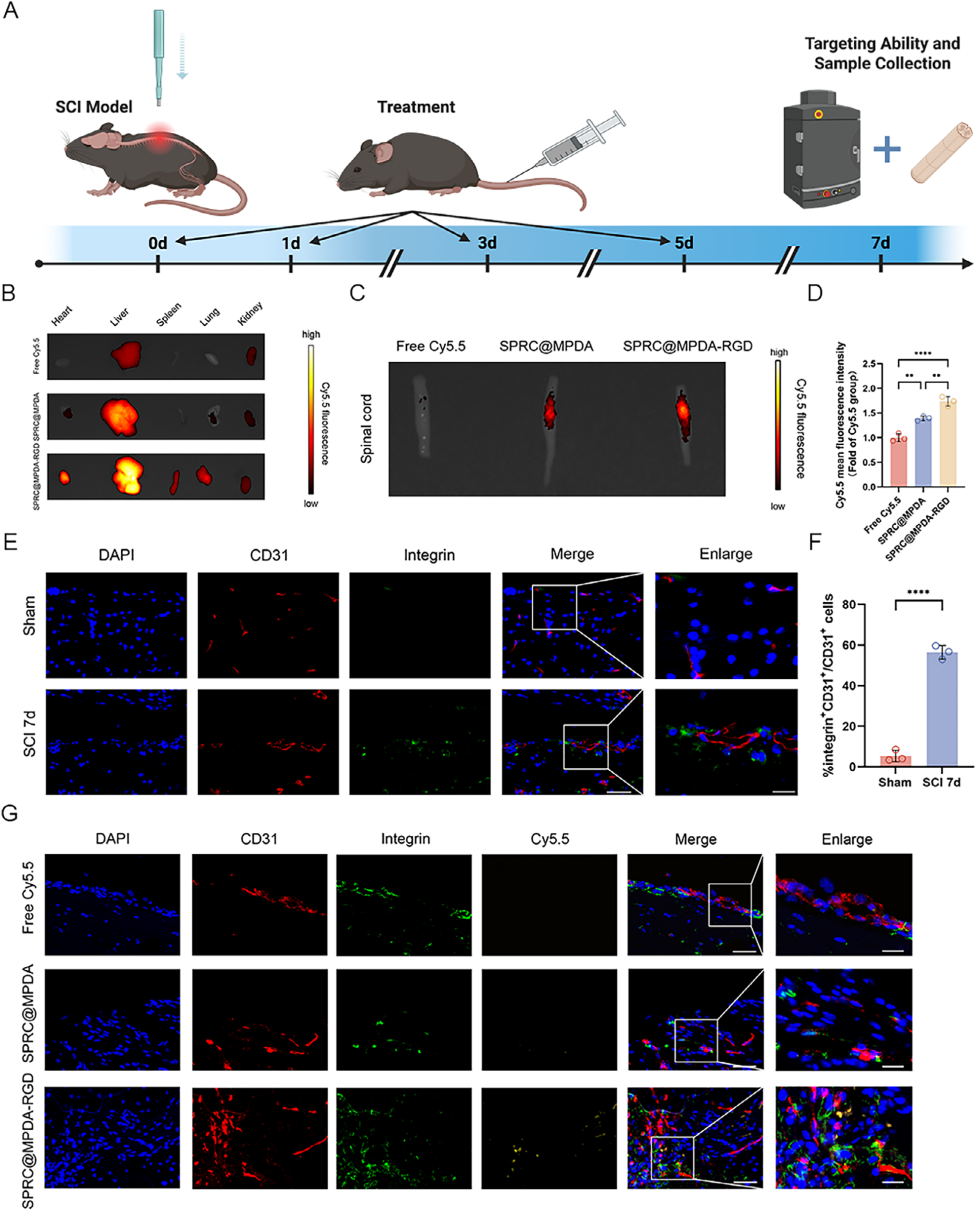
SPRC@MPDA‐RGD specifically targeted vascular endothelial cells at the site of SCI injury. (A) Schematic diagram of the targeting experimental procedure. (B) Ex vivo IVIS images of the mouse heart, liver, spleen, lung, and kidney harvested at 7 days post‐SCI in different groups. (C,D) Representative IVIS images of the spinal cord and quantitative analysis of injury site fluorescence intensity (n = 3). (E,F) Representative immunofluorescence images and quantitative analysis results of integrin α_v_β_3_ in the endothelial cells (marked by CD31) (n = 3). Scale bar = 50 µm; enlarge scale bar = 20 µm. (G) Co‐labeled fluorescence imaging of Cy5.5 (yellow) with integrin α_v_β_3_ (green) and CD31 (red) in spinal cord regions. Scale bar = 50 µm; enlarge scale bar = 20 µm. Data are expressed as mean ± SD. ^**^ *p* < 0.01, ^****^ *p* < 0.0001, one‐way ANOVA with Tukey's multiple comparisons tests (D) and two‐tailed unpaired Student's t‐test (F).

We first examined the targeting properties of SPRC@MPDA‐RGD. We separately labeled SPRC@MPDA and SPRC@MPDA‐RGD with fluorescent dye Cy5.5, and then verified their targeting efficiency using In Vivo Imaging Systems (IVIS) (Figure 3A). At 7 days post‐injury, the results showed that small amounts of SPRC@MPDA could accumulate at the injury site. After c(RGDyK) modification, SPRC@MPDA‐RGD significantly enhanced specific accumulation at the injury site, suggesting that RGD‐modified nanoparticles have enhanced targeting ability to the spinal cord lesion area (Figure 3C,D). We also harvested the main organs and assessed the accumulation of the free Cy5.5, SPRC@MPDA, and SPRC@MPDA‐RGD in them. The results showed that both SPRC@MPDA and SPRC@MPDA‐RGD accumulated mainly in the liver (Figure 3B). To further substantiate the targeting specificity of SPRC@MPDA‐RGD in BSCB, we performed co‐staining of CD31 and integrin α_v_β_3_ on the spinal cord at the injury site. Similar to previous studies [44, 48], the results showed that integrin α_v_β_3_ was mainly expressed on endothelial cells after injury (Figure 3E,F). In addition, co‐fluorescence staining of α_v_β_3_ and CD31 with Cy5.5 revealed that the SPRC@MPDA‐RGD group showed significantly enhanced targeting to integrin‐positive vascular endothelial cells in the damaged area after SCI compared to SPRC@MPDA group (Figure 3G; Figure S4). We also tested the biological safety of SPRC@MPDA‐RGD, and the results showed that SPRC@MPDA‐RGD does not cause biological toxicity (Figure S5A–F).

### 2.4 SPRC@MPDA‐RGD Promoted Spinal Cord Repair and Improved Functional Recovery

**FIGURE 4:**
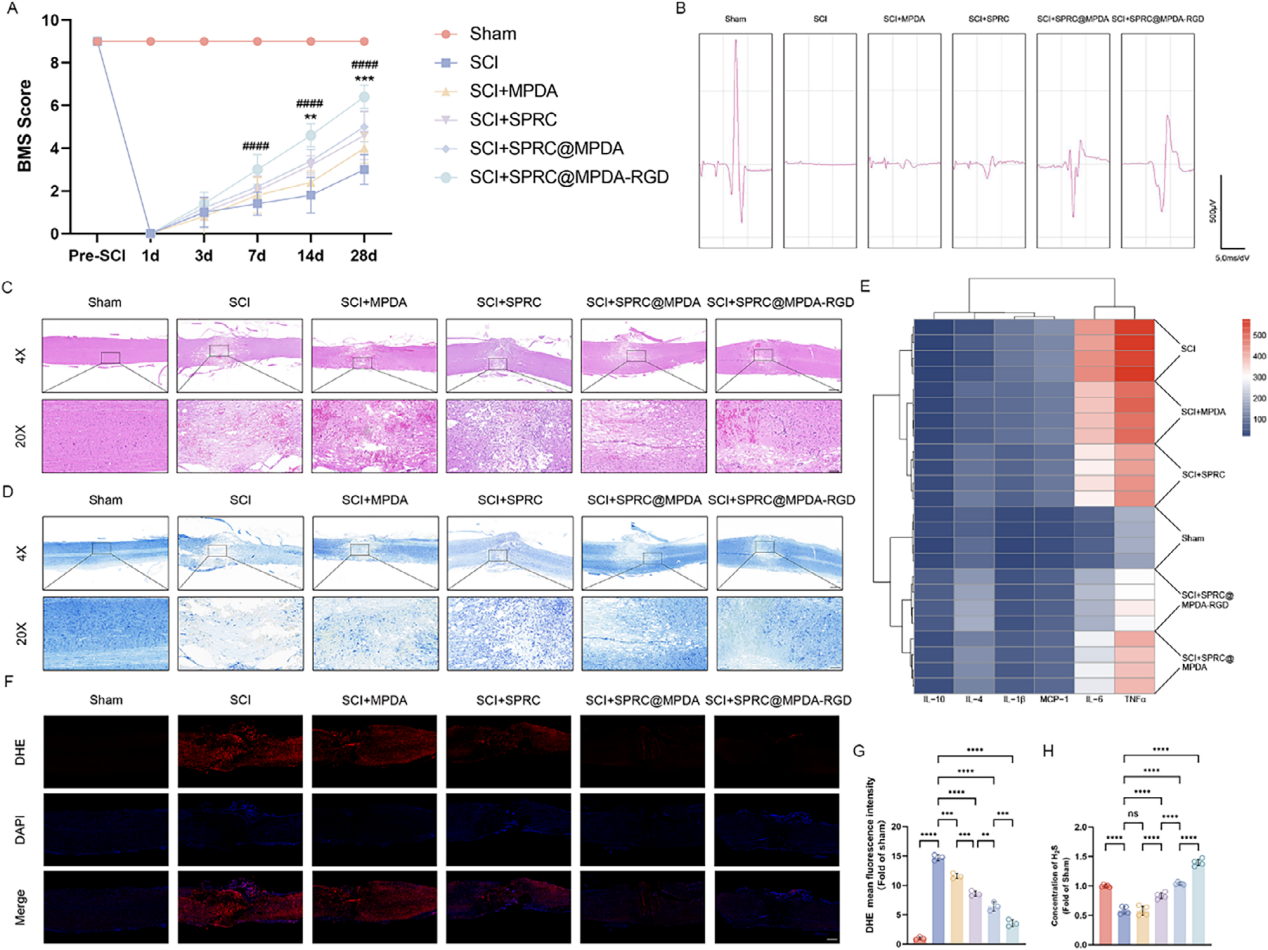
SPRC@MPDA‐RGD promoted functional recovery and mitigated pathology after SCI. (A) Locomotor functional recovery assessed by BMS scores in mice under different treatments. Data are expressed as mean ± SD (n = 5). ^####^ *p* < 0.0001 (vs the SCI group), ^**^ *p* < 0.01 (vs the SCI+SPRC@MPDA group), ^***^ *p* < 0.001 (vs the SCI+SPRC@MPDA group), two‐way ANOVA with the Tukey's multiple comparisons tests. B) Representative electrophysiological images of MEP in mice after different treatments. (C) HE staining of the spinal cord in each group on day 7 post‐injury. 4X Scale bar = 500 µm; 20X Scale bar = 100 µm. (D) Nissl staining of the spinal cord in each group on day 7 post‐injury. 4X Scale bar = 500 µm; 20X Scale bar = 100 µm. (E) Heatmap depicting levels of pro‐inflammatory cytokines (MCP‐1, IL‐6, IL‐1β, and TNF‐α) and anti‐inflammatory cytokines (IL‐4 and IL‐10) in the spinal cord in each group determined by RayPlex^TM^ Mouse Inflammation Array 1 kit. (F,G) Representative staining images and the quantitative analysis results of DHE (n = 3). Scale bar = 500 µm. H) H_2_S concentrations in spinal cord tissue at the injury site in different groups at 7 days after SCI (n = 5). Data are expressed as mean ± SD. ^**^ *p* < 0.01, ^***^ *p* < 0.001, ^****^ *p* < 0.0001, ns means no significance, one‐way ANOVA with Tukey's multiple comparisons tests (G, H).

The SPRC@MPDA‐RGD's therapeutic effect in vivo was evaluated by establishing the SCI model and subsequently administering the SPRC@MPDA‐RGD by tail vein injection. The functional recovery in mice of different groups was evaluated by the BMS score. The results demonstrated that the mice in all groups were immediately paralyzed after SCI, and then exhibited different recovery effects after different interventions (Figure 4A). Compared with the SCI group, mice administered SPRC@MPDA‐RGD showed significant improvement in BMS scores on the seventh day post injury. And the functional recovery of mice treated with SCI+SPRC@MPDA‐RGD improved more significantly than those treated with the SCI+SPRC@MPDA after two weeks post injury. The inclined plane test yielded similar results, demonstrating that SPRC@MPDA‐RGD significantly improved hindlimb motor function in mice after injury, with a more substantial effect than SPRC@MPDA (Figure S6). The results of electrophysiological experiments also demonstrated that the group treated with SPRC@MPDA‐RGD had higher amplitude of MEP signals compared to other treatment groups (Figure 4B). We also performed HE staining and Nissl staining for the spinal cord on 7 days after SCI, which demonstrated that SPRC@MPDA‐RGD significantly reduced both the lesion areas and Nissl bodies loss post SCI in comparison with other groups (Figure 4C,D). In addition, we also collected spinal cord samples from mice and used them to test inflammation‐related cytokines. As shown in the heatmap (Figure 4E), pro‐inflammatory cytokines exhibited a significant increase following SCI, while the expression of them was significantly downregulated after SPRC@MPDA‐RGD intervention. The contents of anti‐inflammatory cytokines decreased after injury, while SPRC@MPDA‐RGD effectively increased their expression. These results demonstrate that SPRC@MPDA‐RGD can effectively improve the inflammatory environment after SCI. Recent studies have highlighted the importance of increased ROS levels in BSCB disruption [30, 45]. To further investigate the antioxidant ability of SPRC@MPDA‐RGD in vivo, we used DHE staining to assess the oxidative stress statement within the spinal cord. The results showed that SPRC@MPDA‐RGD was most effective in reducing ROS levels in the spinal cord following injury (Figure 4F,G). And we also tested the H_2_S content in spinal cord tissue within different treatment groups. As expected, the content decreased significantly after SCI, but SPRC@MPDA‐RGD effectively reversed it (Figure 4H). It is noteworthy that SPRC@MPDA‐RGD exhibited statistical superiority over SPRC@MPDA in enhancing motor function recovery, improving inflammatory environment, mitigating oxidative stress, and restoring H_2_S levels. Moreover, electrophysiological experiments and pathological examinations also demonstrated that SPRC@MPDA‐RGD was more effective than SPRC@MPDA after SCI in promoting recovery. These results suggest that c(RGDyK) significantly improves the delivery efficiency of SPRC@MPDA, thereby enhancing therapeutic effect following injury.

### 2.5 SPRC@MPDA‐RGD Alleviated Blood‐Spinal Cord Barrier Destruction In Vivo after Injury

**FIGURE 5:**
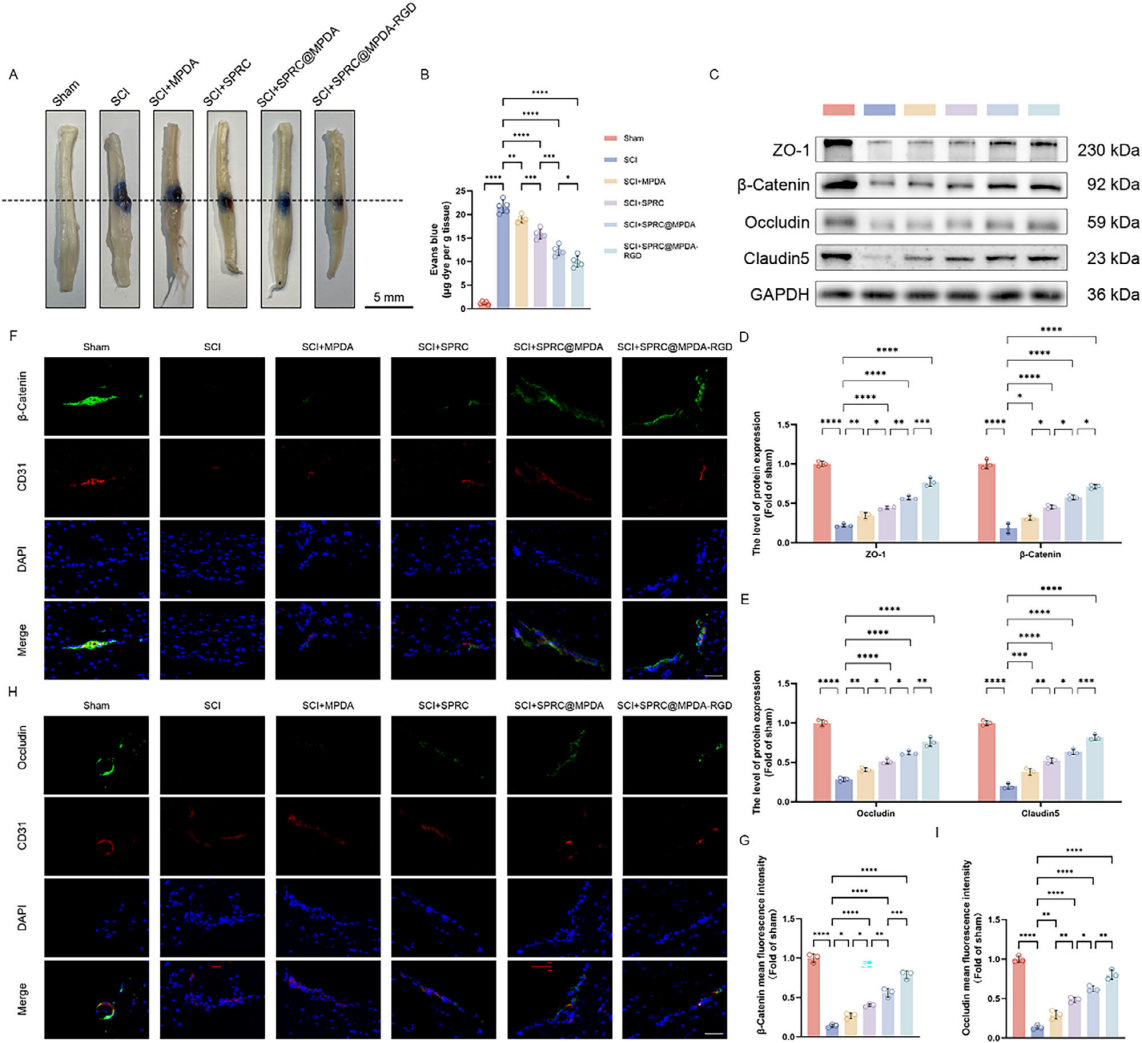
SPRC@MPDA‐RGD protected the integrity of BSCB. (A,B) Representative spinal cord images of EB assay and quantitative analysis results of EB staining in mice between different groups (n = 5). Scale bar = 5 mm. (C) Western blotting showed the expression level of BSCB‐related protein (ZO‐1, β‐Catenin, Occludin, and Claudin5) in the spinal cord segment at the injury epicenters. (D,E) Quantitative analysis of ZO‐1, β‐Catenin, Occludin, and Claudin5 protein expression (n = 3). (F,G) Representative immunofluorescence images and the quantitative analysis results of β‐Catenin in the endothelial cells (marked by CD31) (n = 3). Scale bar = 50 µm. (H,I) Representative immunofluorescence images and the quantitative analysis results of Occludin in the endothelial cells (marked by CD31) (n = 3). Scale bar = 50 µm. Data are expressed as mean ± SD. ^*^ *p* < 0.05, ^**^ *p* < 0.01, ^***^ *p* < 0.001, ^****^ *p* < 0.0001, one‐way ANOVA with Tukey's multiple comparisons tests.

Recent studies have shown that SPRC has the capacity to enhance endothelial cell function, including cell proliferation and cell viability through increasing the CSE expression in endothelial cells to produce endogenous H_2_S [24, 25]. Our in vitro studies have also demonstrated that SPRC@MPDA‐RGD can improve BSCB‐related dysfunction. Therefore, examinations of BSCB‐related functions in mice from different treatment groups were conducted after SCI. The Evans blue (EB) assay presented that SPRC@MPDA‐RGD effectively attenuated injury‐induced elevation of BSCB permeability (Figure 5A,B). Furthermore, spinal cord samples were collected on the seventh day after injury, and then western blotting was conducted to assess the differences of BSCB‐related proteins between groups. The results demonstrated that SPRC@MPDA‐RGD effectively alleviated the loss of BSCB‐related proteins caused by SCI and restored their expression, including β‐Catenin, ZO‐1, Claudin5, and Occludin (Figure 5C–E). The results of the double‐labeling immunofluorescence analysis of β‐Catenin and Occludin further corroborated these findings (Figure 5F–I). In summary, SPRC@MPDA‐RGD can improve BSCB function after SCI and provide effective protection.

### 2.6 Transcriptome Alteration of Spinal Cord Injury following SPRC@MPDA‐RGD Treatment

**FIGURE 6:**
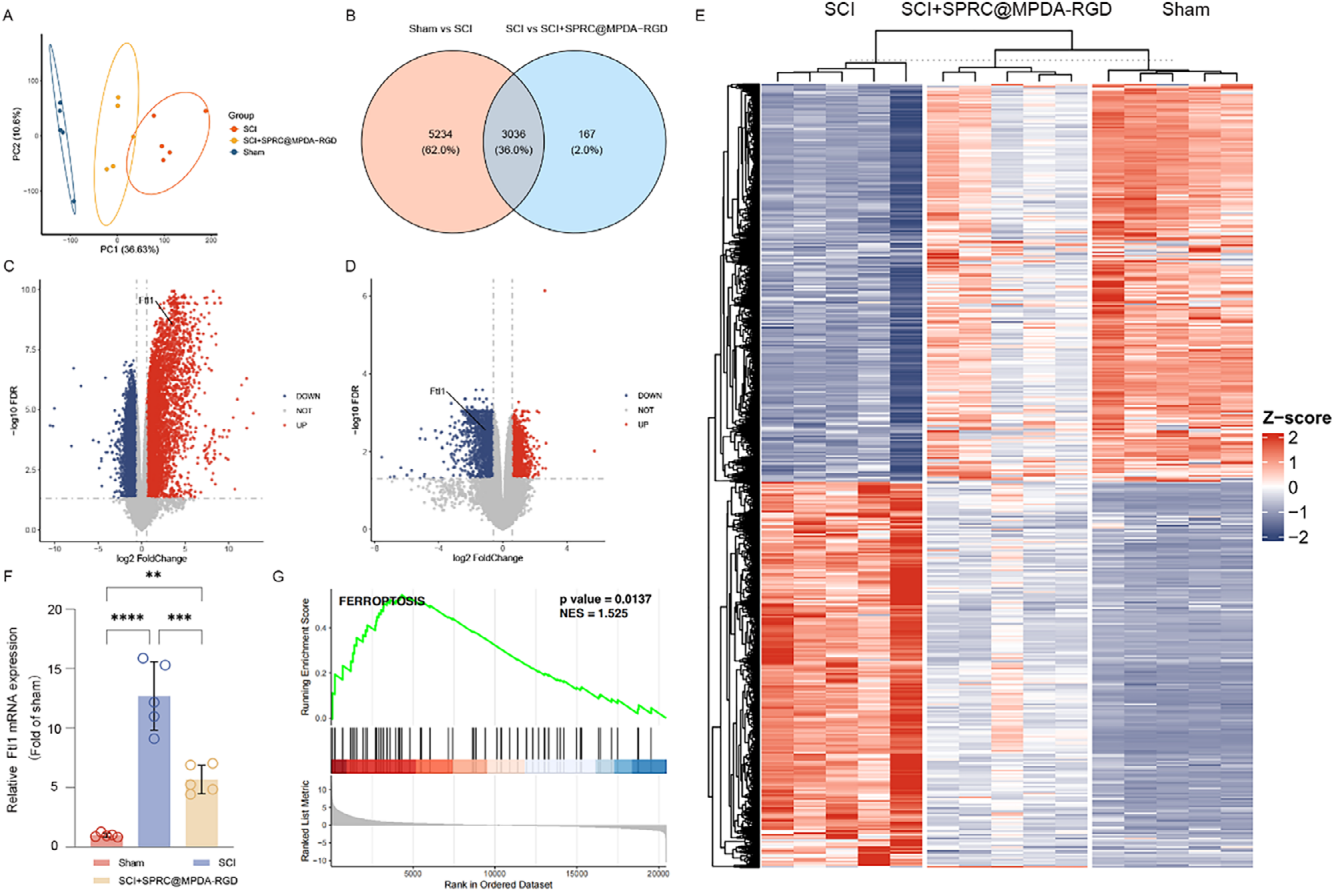
RNA‐seq revealed differential gene expression and pathway modulation after treatment in mice after SCI, SCI+SPRC@MPDA‐RGD, and Sham.)(A) PCA of transcriptomics in mice after SCI, SCI+SPRC@MPDA‐RGD, and Sham. (B) Venn diagram shows the overlap of DEGs between two comparisons: Sham versus SCI and SCI versus SCI + SPRC@MPDA‐RGD. Shared genes represent those altered by spinal cord injury (SCI) and modulated by SPRC@MPDA‐RGD treatment. (C) Volcano plot of DEGs for SCI and Sham comparison. Genes with FDR < 0.05 and absolute log2 fold change >0.585 are considered as DEGs. (D) Volcano plot of DEGs for SCI+SPRC@MPDA‐RGD and SCI comparison. Genes with FDR < 0.05 and absolute log2 fold change >0.585 are considered as DEGs. E) The heatmap of the intersection of DEGs between SCI and Sham, and between SCI+SPRC@MPDA‐RGD and SCI. (F) Bar plot shows the expression level of Ftl1 from RNA‐seq data, presented as fold change relative to the Sham group (n = 5). (G) Gene Set Enrichment Analysis (GSEA) on the ranked list of genes in the “WP_FERROPTOSIS” pathway. Data are expressed as mean ± SD. ^**^ *p* < 0.01, ^***^ *p* < 0.001, ^****^ *p* < 0.0001, one‐way ANOVA with Tukey's multiple comparisons tests.

To explore the differentially expressed genes (DEGs) to SCI and investigate the molecular mechanisms underlying the effect of SPRC@MPDA‐RGD treatment, we performed bulk RNA‐seq on spinal cord samples from mice in Sham, SCI, and SCI+SPRC@MPDA‐RGD groups. Principal component analysis (PCA) revealed clear separation among the Sham, SCI, and SCI+SPRC@MPDA‐RGD groups, with PC1 accounting for 36.63% and PC2 accounting for 10.6% of the total variance (Figure 6A). The SCI group clustered separately from the Sham group, whereas the SCI+SPRC@MPDA‐RGD group localized between the two groups, indicating that the treatment of SPRC@MPDA‐RGD shifted the transcriptomic landscape toward a recovered state. The Venn diagram presents the overlap of DEGs between the comparisons of Sham versus SCI and SCI versus SCI + SPRC@MPDA‐RGD (Figure 6B). A total of 5,234 genes (62.0%) were uniquely differentially expressed in the Sham versus SCI, indicating gene expression changes specifically associated with SCI, while 167 genes (2.0%) were uniquely altered in the SCI versus SCI + SPRC@MPDA‐RGD comparison, suggesting potential gene targets specifically modulated by SPRC@MPDA‐RGD treatment. The results of the GO and KEGG enrichment analyses on these 167 unique genes involve endothelial cell proliferation, suggesting that SPRC@MPDA‐RGD may exert a protective effect on the BSCB after SCI by regulating endothelial cell function and promoting the recovery of neurological function (Figure S7A,B). Importantly, 3036 genes (36.0%) were shared between the two comparisons, representing genes that are dysregulated in SCI and subsequently influenced by the treatment. The volcano plot comparing the SCI and Sham groups showed substantial upregulation of 3237 genes and downregulation of 2158 genes, indicating the significant impact of SCI on gene expression (Figure 6C). In contrast, comparison between the SCI+SPRC@MPDA‐RGD and SCI groups revealed significant changes in 594 upregulated genes and 834 downregulated genes (Figure 6D). The heatmap showed the intersection of DEGs between SCI and Sham, and between SCI+SPRC@MPDA‐RGD and SCI. The SCI group exhibited widespread gene expression changes compared to the Sham group, while the SCI+SPRC@MPDA‐RGD group displayed a partially restored gene expression pattern, suggesting a therapeutic modulation of injury‐induced transcriptional dysregulation (Figure 6E). The heatmap of genes involved in oxidative and inflammatory pathway showed similar result. With the treatment of SPRC@MPDA‐RGD, the widespread gene expression changes caused by SCI were also partially restored (Figure S8A,B). Ferritin, the principal iron storage protein, fulfils a pivotal role in iron homeostasis by means of storing and releasing iron ions [49, 50]. Structurally, ferritin consists of two subunits, the ferritin light chain (FTL) and the ferritin heavy chain (FTH), which are encoded by the Ftl1 and Fth1 genes respectively and finally forming the apoferritin shell [51, 52]. When free excess iron is not properly stored in ferritin cores, it often leads to oxidative stress, ultimately resulting in lipid peroxidation and ferroptosis [53]. In our study, Ftl1 mRNA expression, was found upregulated in the SCI group compared with Sham group, and was restored in the SCI+SPRC@MPDA‐RGD group (Figure 6F). Therefore, we analyzed the ferroptosis pathway using Gene Set Enrichment Analysis (GSEA) based on the ranked gene list. The results suggest that the ferroptosis pathway is significantly enriched after SCI, and the therapeutic effect of SPRC@MPDA‐RGD may be related to ferroptosis (Figure 6G). Together, these findings suggest that SPRC@MPDA‐RGD treatment can significantly alter the transcriptional response to SCI and potentially mediate protective effects on BSCB by modulating key injury‐related gene networks.

### 2.7 SPRC@MPDA‐RGD Alleviated Ferroptosis in Endothelial Cells Caused by OGD

**FIGURE 7:**
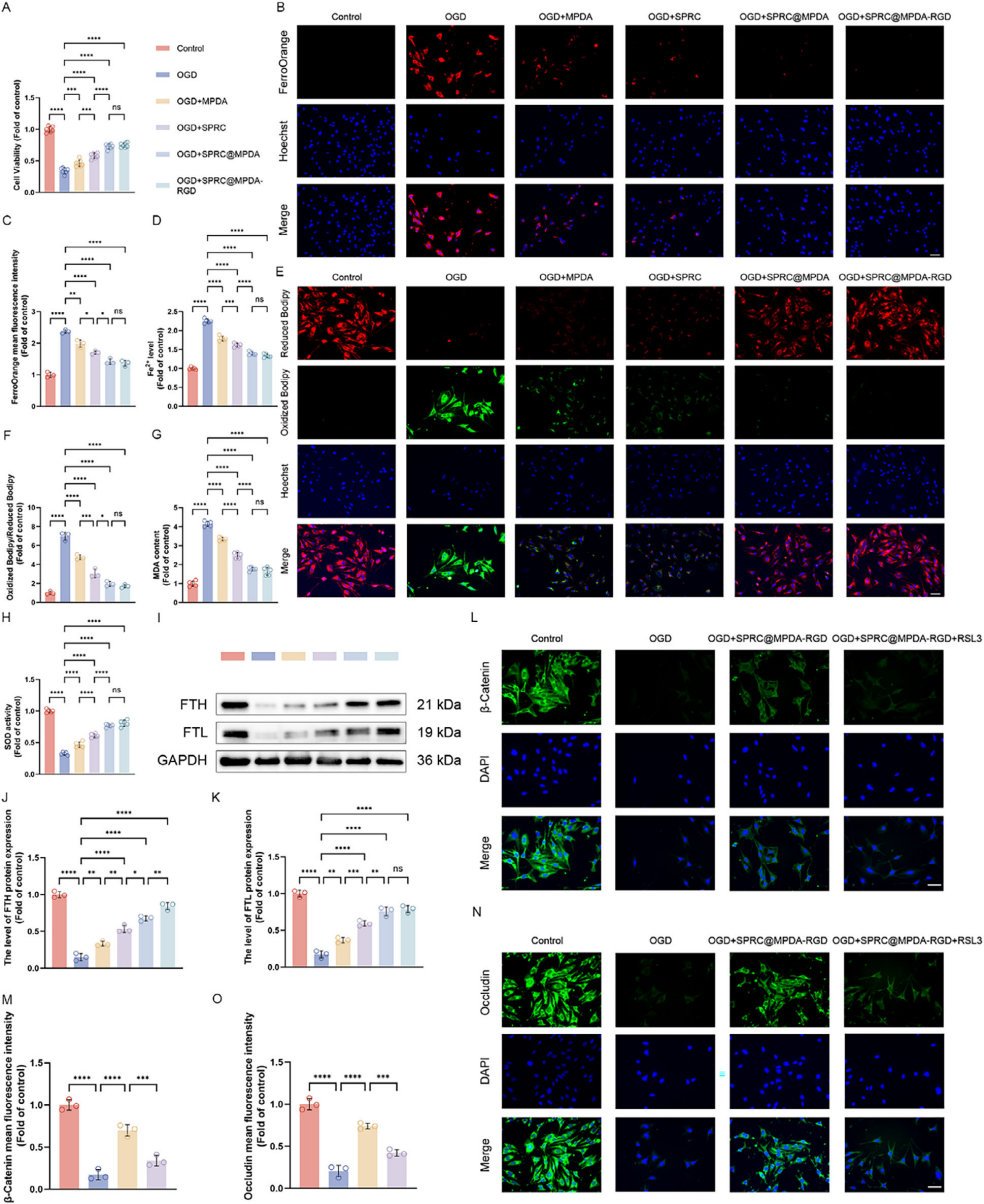
SPRC@MPDA‐RGD inhibited ferroptosis in bEnd.3 cells induced by OGD. (A) The cell viability detected by CCK‐8 assay after OGD and different treatments (n = 7). (B,C) Representative images and quantitative analysis results of intracellular Fe^2+^ levels in bEnd.3 cells assessed using FerroOrange fluorescent probe (n = 3). Scale bar = 50 µm. (D) Quantification of intracellular Fe^2+^ content in different groups assessed by The Ferrous Iron Colorimetric Assay Kit (n = 5). (E,F) Representative images and quantitative analysis results of intracellular lipid ROS levels assessed by C11‐BODIPY probe (n = 3). Scale bar = 50 µm. (G) MDA content levels in bEnd.3 cells (n = 5). (H) SOD activity in bEnd.3 cells in different groups (n = 5). (I) Western blotting showed the expression level of FTH and FTL in bEnd.3 cells. (J,K) Quantitative analysis of FTH and FTL protein expression (n = 3). (L,M) Representative immunofluorescence images and the quantitative analysis results of β‐Catenin expression in bEnd.3 cells after different treatments (n = 3). Scale bar = 50 µm. (N,O) Representative immunofluorescence images and the quantitative analysis results of Occludin expression in bEnd.3 cells after different treatments (n = 3). Scale bar = 50 µm. Data are expressed as mean ± SD. ^*^ *p* < 0.05, ^**^ *p* < 0.01, ^***^ *p* < 0.001, ^****^ *p* < 0.0001, ns means no significance, one‐way ANOVA with Tukey's multiple comparisons tests.

As demonstrated in our previous research, ferroptosis in endothelial cells following injury is a significant contributing factor to endothelial cell dysfunction [16]. However, it remains unknown whether SPRC@MPDA‐RGD protects BSCB by alleviating ferroptosis in endothelial cells. Subsequent to the results of bulk RNA‐seq, indicators associated with ferroptosis in endothelial cells under different interventions were further tested. We first detected the cell viability of endothelial cells in different groups. The OGD significantly reduced the bEnd.3 viability, while SPRC@MPDA‐RGD exhibited a substantial capacity to restore it (Figure 7A). This ability was analogous to that of SPRC@MPDA and significantly superior to that of other single components. Subsequently, two important hallmarks of ferroptosis were evaluated: the levels of labile ferrous ion (Fe^2+^) and lipid peroxidation. The Ferrous Iron Colorimetric Assay Kit and FerroOrange fluorescent staining were utilized to evaluate intracellular Fe^2+^ levels in bEnd.3 cells. As expected, after OGD intervention, there was a substantial increase in the level of Fe^2+^ within the bEnd.3 (Figure 7B–D). Both MPDA and SPRC were capable of moderately reducing Fe^2+^ levels and exerting synergistic effects, with the SPRC@MPDA‐RGD group demonstrating the most significant reduction in Fe^2+^ levels. The lipid peroxidation levels were detected by C11‐BODIPY probe in different groups. The lipid ROS levels increased after OGD, while SPRC@MPDA‐RGD significantly alleviated this (Figure 7E,F). Furthermore, the evaluation of key ferroptosis indicators, including superoxide dismutase (SOD, an enzyme could inhibit ROS), and malondialdehyde (MDA, lipid peroxidation's end product), was also conducted. The results demonstrated that SOD activity was decreased and MDA content levels were elevated in the OGD group (Figure 7G,H). However, SPRC@MPDA‐RGD significantly mitigated this process. In summary, SPRC@MPDA‐RGD can effectively alleviate ferroptosis in endothelial cells caused by OGD. It is noteworthy that single‐component MPDA or SPRC also exhibit moderate ferroptosis inhibitory activity, which may be attributed to MPDA's chelating ability for Fe ions and the inhibitory effect of H_2_S released by SPRC on ferroptosis in endothelial cells respectively [17, 34]. The results demonstrate that these two components can exert synergistic effects, and the SPRC@MPDA‐RGD composite exhibits potent ferroptosis inhibitory activity. Finally, the expression of FTH and FTL was determined by western blotting among different groups. The results demonstrated a significant decrease in both FTH and FTL following OGD, thereby indicating a substantial reduction in ferritin and the release of iron into the labile iron pool, potentially leading to iron overload (Figure 7I–K) [54]. This may be a significant factor contributing to the ferroptosis in endothelial cells induced by OGD.

To determine whether the BSCB protection by SPRC@MPDA‐RGD was mediated through the inhibition of ferroptosis, we conducted further investigations. Using the ferroptosis inducer RSL3, SPRC@MPDA‐RGD's protective effect on BSCB can be reversed by RSL3, demonstrating that its therapeutic effect is achieved through the inhibition of ferroptosis (Figure 7L–O).

### 2.8 SPRC@MPDA‐RGD Inhibited OGD‐Induced Ferritinophagy

**FIGURE 8:**
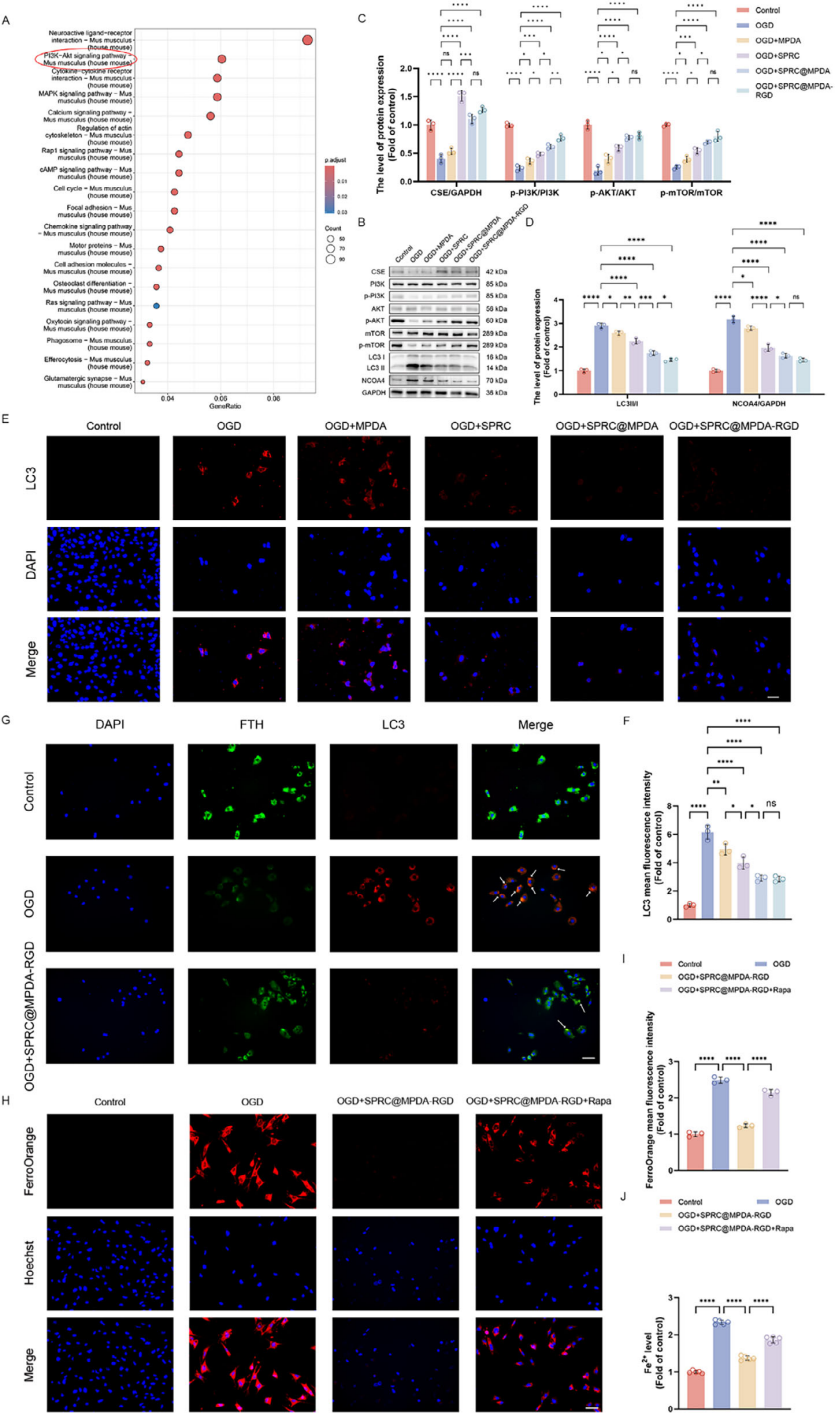
SPRC@MPDA‐RGD inhibited ferritinophagy to alleviate ferroptosis in bEnd.3 cells. (A) Bubble plot of KEGG enrichment analysis showed top enriched KEGG pathways based on the DEGs between SCI and Sham, and between SCI+SPRC@MPDA‐RGD and SCI. The x‐axis indicates the GeneRatio, the ratio of genes enriched in a pathway over the total number of genes in the set. The size of the bubble represents the gene count. The color gradient representing the adjusted p‐value. Significantly enriched KEGG pathways are shown (Adjusted *p*‐value by Benjamini‐Hochberg < 0.05). (B) Western blotting showed the expression level of CSE, PI3K, p‐PI3K, AKT, p‐AKT, mTOR, p‐mTOR, LC3II, LC3I, and NCOA4 of bEnd.3 cells in different groups. (C,D) Quantitative analysis of CSE, p‐PI3K/ PI3K, p‐AKT/AKT, p‐mTOR/ mTOR, LC3II/LC3I and NCOA4 protein expression (n = 3). (E,F) Representative immunofluorescence images and the quantitative analysis results of LC3 (n = 3). Scale bar = 50 µm. (G) Colocalization of FTH (green signal) and LC3 (red signal) in bEnd.3 cell in different groups. Note that the white arrows indicated possible colocalization of FTH and LC3. Scale bar = 50 µm. (H,I) Representative images and quantitative analysis results of intracellular Fe^2+^ levels in bEnd.3 cells assessed using FerroOrange fluorescent probe (n = 3). Scale bar = 50 µm. (IJ) The levels of intracellular Fe^2+^ content in different groups assessed by The Ferrous Iron Colorimetric Assay Kit (n = 5). Data are expressed as mean ± SD. ^*^ *p* < 0.05, ^**^ *p* < 0.01, ^***^ *p* < 0.001, ^****^ *p* < 0.0001, ns means no significance, one‐way ANOVA with Tukey's multiple comparisons tests.

Ferritin has been shown to participate in ferritinophagy and cellular iron delivery [51, 53]. KEGG pathway analysis was performed based on bulk RNA‐seq in order to further investigate the potential molecular mechanisms by which SPRC@MPDA‐RGD regulates ferroptosis. The SPRC@MPDA‐RGD‐responsive genes are enriched in the PI3K‐Akt signaling (Figure 8A). As a classic pathway regulating autophagy, it has been shown to inhibit this process by targeting the downstream target mTOR following activation [55]. Previous study has shown that H_2_S exerts a protective effect on neurological recovery through the activation of the PI3K/Akt/mTOR pathway and thus inhibits autophagy [14]. These results suggest that SPRC@MPDA‐RGD's capacity to inhibit ferroptosis may be accomplished via PI3K/Akt/mTOR‐mediated autophagy regulation. Ferritinophagy is a recently unveiled selective type of autophagy that is crucial for connecting autophagy and ferroptosis pathways. Under the regulation of nuclear receptor coactivator 4 (NCOA4), ferritin can be transported to lysosomes and degraded, releasing iron into labile iron pools and triggering ferroptosis [54, 56, 57]. This process is critical in maintaining iron homeostasis. First, the protein expression level of CSE was detected. As expected, MPDA did not contribute to the activation of CSE, while SPRC effectively increased the CSE expression level in bEnd.3 (Figure 8B,C). The results of western blotting in FTH and FTL expression suggested that ferritin may have undergone degradation, and SPRC@MPDA‐RGD may protect the BSCB function through ferritinophagy. Therefore, the proteins participating in autophagy were detected (Figure 8B–D). The results demonstrated that following OGD, there was a significant inhibition of the PI3K/Akt/mTOR signaling, accompanied by substantial activation of autophagy. Furthermore, the expression of NCOA4 was found to be significantly increased after OGD. Considered with the decline in FTL and FTH expression after OGD, these results indicate that ferritinophagy can be significantly induced by OGD. Following treatment with SPRC@MPDA‐RGD, MPDA and SPRC exerted synergistic effects, significantly activating the PI3K/Akt/mTOR signaling, effectively inhibiting autophagy, and reducing NCOA4 expression, indicating that ferritinophagy in bEnd.3 cells was effectively alleviated. Immunofluorescence revealed similar results, with OGD significantly activating autophagy in bEnd.3 cells, while SPRC@MPDA‐RGD inhibited excessive autophagy activation (Figure 8E,F). Additionally, the immunofluorescence colocalization analysis of LC3 and FTH demonstrated that following OGD, FTH and LC3 co‐localization significantly increased, while SPRC@MPDA‐RGD can significantly reverse this process (Figure 8G). This demonstrates that FTH undergoes degradation via autophagy following OGD, while SPRC@MPDA‐RGD effectively inhibits this process. To further validate the protective effect of SPRC@MPDA‐RGD via inhibiting ferritinophagy, bEnd.3 cells were treated with the autophagy inducer rapamycin (Rapa). As expected, SPRC@MPDA‐RGD significantly reduced Fe^2+^ levels, but this ability could be reversed by Rapa (Figure 8H–J). In addition, the ability of SPRC@MPDA‐RGD to alleviate lipid peroxidation by reducing the lipid ROS levels can also be significantly reversed by Rapa (Figure S9A,B). Our results also indicated that the ability of SPRC@MPDA‐RGD to alleviate ferroptosis by reducing MDA content and increasing SOD activity can also be reversed by co‐treatment with Rapa (Figure S9C,D). The results underscore the critical involvement of ferritinophagy in mediating the anti‐ferroptotic and cytoprotective actions of SPRC@MPDA‐RGD.

### 2.9 SPRC@MPDA‐RGD Alleviated Ferroptosis after Injury by Suppressing the Ferritinophagy Pathway

**FIGURE 9:**
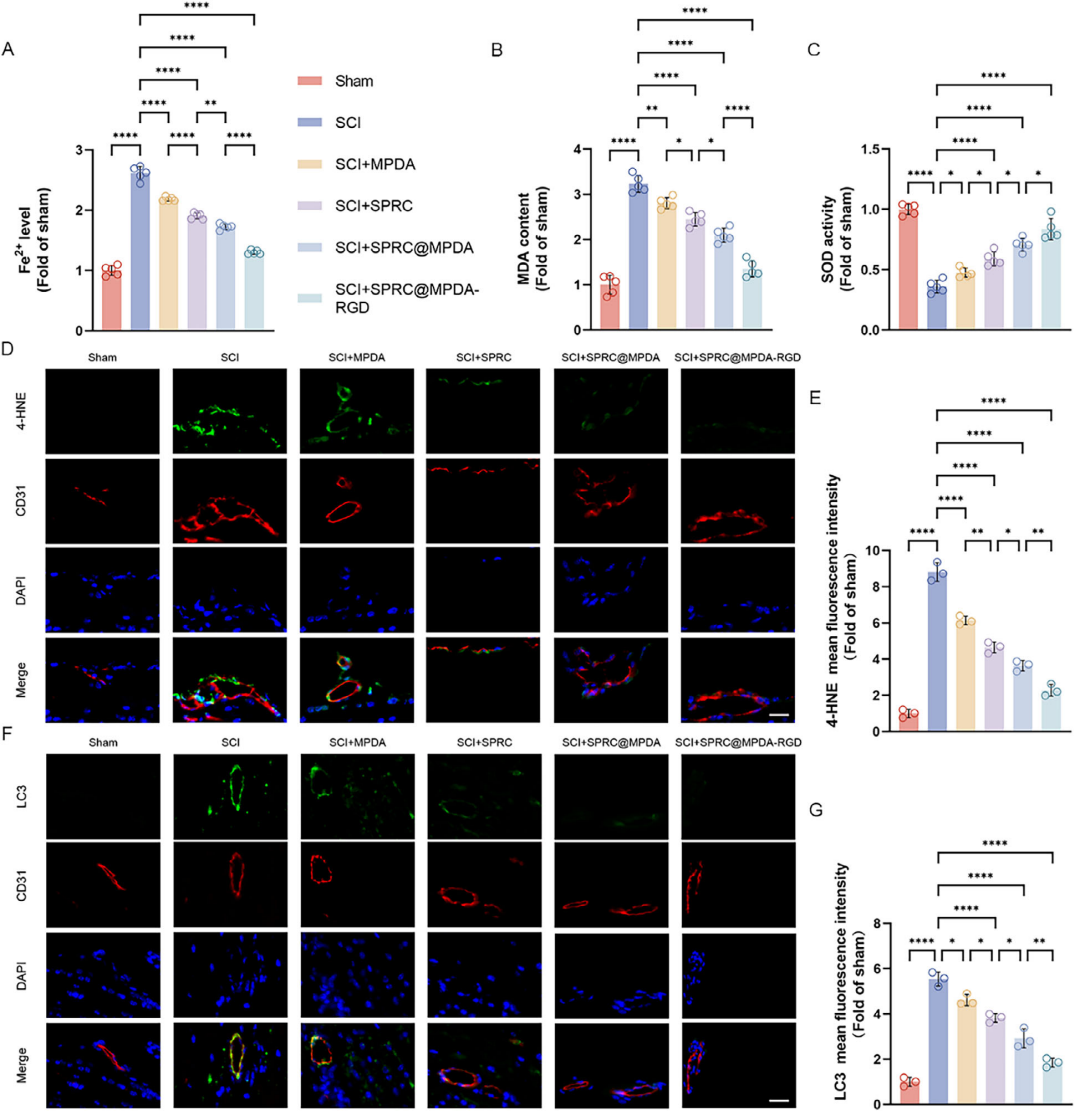
SPRC@MPDA‐RGD inhibited ferritinophagy after SCI. (A) The level of Fe^2+^ content in different groups of spinal cord tissue at 7 days after SCI (n = 5). (B) MDA content levels in spinal cord tissue from mice in different groups (n = 5). (C) SOD activity in spinal cord tissue from mice treated with different interventions (n = 5). (D,E) Representative immunofluorescence images and the quantitative analysis results of 4‐HNE in the endothelial cells (marked by CD31) (n = 3). Scale bar = 50 µm. (F,G) Representative immunofluorescence images and the quantitative analysis results of LC3 in the endothelial cells (marked by CD31) (n = 3). Scale bar = 50 µm. Data are expressed as mean ± SD. ^*^ *p* < 0.05, ^**^ *p* < 0.01, ^****^ *p* < 0.0001, one‐way ANOVA with Tukey's multiple comparisons tests.

We then conducted further in vivo experiments to investigate the protective mechanism of SPRC@MPDA‐RGD. We first tested the concentration of Fe^2+^ and found that it had increased significantly following SCI, while SPRC@MPDA‐RGD had the ability to reduce it (Figure 9A). Furthermore, MDA increased significantly while SOD activity was inhibited in the spinal cord following SCI. Treatment with SPRC@MPDA‐RGD effectively alleviated this process (Figure 9B,C). Following injury, the spinal cord exhibited a significant increase in the lipid peroxidation product 4‐HNE, which was significantly reduced by SPRC@MPDA‐RGD (Figure 9D,E). In summary, the results indicate that SPRC@MPDA‐RGD can effectively alleviate ferroptosis caused by SCI. The levels of LC3 were also detected in the spinal cords after different treatments. The immunofluorescence results demonstrated that autophagy was significantly activated after SCI, and SPRC@MPDA‐RGD could effectively inhibit it (Figure 9F,G). This suggested that SPRC@MPDA‐RGD exerts its inhibitory effect on ferroptosis induced by SCI via the ferritinophagy pathway.

## 3 Discussion

The BSCB, a specialized interface between the circulatory system and the CNS, crucially maintains CNS homeostasis by restricting entry of peripheral immune cells, toxins, pathogens, and inflammatory mediators under physiological conditions [58, 59]. The treatment of SCI remains limited, where disruption of the BSCB serves as a critical nexus for secondary pathogenesis. Following SCI, disruption of endothelial tight junctions (e.g., ZO‐1, Claudin‐5, Occludin) and adherens junctions (e.g., β‐catenin) compromises BSCB integrity, resulting in increased vascular permeability. This permits infiltration of cytokines and inflammatory cells into the injured parenchyma, driving progressive secondary injury [59, 60]. Consequently, early BSCB repair represents a critical therapeutic objective in SCI management.

We engineered SPRC@MPDA‐RGD nanoparticles for spatially controlled H_2_S delivery. Unlike conventional inorganic H_2_S donors that cause cytotoxic bursts [21, 61], SPRC@MPDA‐RGD leverages endogenous enzymatic H_2_S synthesis. By upregulating CSE, the released SPRC promotes sustained, physiological H_2_S levels within endothelial cells, avoiding the toxicity pitfalls of exogenous donors. The resultant combinatorial effect, including targeted H_2_S generation, ROS neutralization, and ferritinophagy inhibition together underlies the robust BSCB repair observed, evidenced by restored junctional proteins (Occludin, ZO‐1, β‐Catenin, and Claudin‐5), reduced vascular leakage, attenuated inflammation, and improved motor function.

Our findings extend the understanding of H_2_S‐mediated vascular protection into the CNS microvasculature [62, 63, 64], revealing its capacity to mitigate post‐SCI oxidative stress and restore BSCB junctional proteins ‐consistent with H_2_S‐mediated endothelial barrier preservation in pulmonary systems [65]. The SPRC@MPDA‐RGD design offers dual advantages: (1) SPRC bypasses the uncontrolled release of inorganic donors by leveraging physiological CSE activation, and (2) MPDA's ROS‐scavenging capability synergizes with SPRC's therapeutic actions [66]. The mesoporous architecture further enables sustained SPRC release, thus effectively avoiding the problem of SPRC with rapid elimination and metabolism in vivo. Like other small‐molecule drugs, SPRC also lacks the ability to target tissues, and the precise delivery of SPRC to vascular endothelia in the spinal cord after injury remains an unresolved issue [29]. To overcome inherent tissue‐targeting deficiencies of SPRC, we exploited post‐SCI upregulation of integrin α_v_β_3_ on neovascular endothelia [44]. Given that specific peptide ligand c(RGDyK) has been shown to bind to integrin α_v_β_3_ with high specificity [40, 41], conjugation of the high‐affinity ligand c(RGDyK) conferred precise endothelial targeting, significantly enhancing lesion‐site accumulation of SPRC@MPDA‐RGD. This targeting paradigm holds broad translational relevance for enhancing the delivery efficiency of BSCB‐directed therapeutics while minimizing off‐target effects. The RGD‐mediated therapeutic strategy is supposed to be employed to enhance the efficacy of other nanomedicines that target BSCB following SCI.

Mechanistically, we identified ferritinophagy‐a ferroptosis process mediated by NCOA4‐as a key pathway in post‐SCI endothelial dysfunction. The ferritinophagy pathway, as a recently unveiled selective autophagy, elucidates how ferroptosis occurs as an autophagic cell death process [67]. Ferritin, which is involved in iron storage, can be transported to the lysosome and degraded through ferritinophagy pathway under the mediation of NCOA4, leading to an increase in intracellular Fe^2+^ levels and ultimately inducing ferroptosis [57]. Recent studies have demonstrated that ROS can induce endothelial dysfunction by triggering ferritinophagy, thereby promoting ferroptosis and ultimately leading to vascular injury [68]. Furthermore, ferritinophagy is also crucial for the inflammatory response of endothelial cells [69, 70]. In atherosclerosis, ferritinophagy has been demonstrated to be a key pathway mediating aortic endothelial inflammation [70]. In SCI, the inhibition of ferroptosis has been demonstrated to effectively mitigate the disruption of BSCB [71]. However, it remains unclear whether this process is regulated by autophagy. Our results demonstrate that SPRC@MPDA‐RGD activates the PI3K/Akt/mTOR axis, suppressing NCOA4‐mediated ferritinophagy in injured endothelial cells. This aligns with prior evidence of H_2_S‐mediated neuroprotection via PI3K/Akt/mTOR activation in traumatic brain injury [14], while revealing novel iron‐homeostasis regulation in CNS microvasculature. Notably, the changes in FTH and FTL protein levels in cellular experiments appeared opposite to the trend of Ftl1 mRNA expression from bulk RNA‐seq. This discrepancy may be because bulk RNA‐seq was performed on whole spinal cord samples, where endothelial cells constitute a minor proportion. The MPDA component likely contributes through ROS scavenging and Fe ions chelation [34, 35], though detailed mechanistic interplay warrants further investigation. This study sheds light on ferritinophagy activation in spinal microvascular endothelia post‐SCI, establishing its modulation as a viable strategy for BSCB protection with implications across vascular pathologies [70, 72].

Despite promising outcomes, several aspects require further investigation: (1) Long‐term biosafety, metabolic characteristics, and functional recovery profiles beyond the acute phase; (2) Elucidation of regulators linking H_2_S with PI3K/Akt/mTOR activation; (3) Validation in higher‐order species (e.g., non‐human primates) is required to confirm the ability to target endothelial cells in higher species post‐injury and to assess clinical translatability, given the interspecies differences in the neurovascular system; (4) Development of strategies for large‐scale batch production and standardized quality controls to enable widespread clinical application must also be considered.

In summary, we have developed an intravenously administered, endothelial‐targeted nanotherapeutic (SPRC@MPDA‐RGD) that leverages α_v_β_3_ integrin overexpression for site‐specific BSCB delivery. This system orchestrates endogenous H_2_S generation via CSE upregulation, synergized by MPDA's antioxidant activity, to effectively restore barrier integrity and functional recovery. Crucially, we identify NCOA4‐driven ferritinophagy as a key driver of endothelial ferroptosis and demonstrate that H_2_S activates the PI3K/Akt/mTOR signaling to suppress this process, thus preserving endothelial iron homeostasis. This work not only substantiates the therapeutic value of H_2_S in SCI and establishes ferritinophagy modulation as a promising strategy for CNS vascular repair, but also fundamentally advances our understanding of BSCB pathophysiology by defining ferritinophagy.

## 4 Experimental Section

### 4.1 Chemicals and Materials

Dopamine hydrochloride and 1, 3, 5‐Trimethylbenzene (TMB) were bought from Aladdin Reagent (Shanghai, China). Ammonia aqueous solution (NH_3_·H_2_O, 30 wt %) was obtained from Guangzhou Chemical Reagent Factory (Guangzhou, China). c(RGDyK) was purchased from GL Biochem Ltd. (Shanghai, China). Pluronic F127 was obtained from Sigma‐Aldrich (MO, USA). 1,3‐Dimethyl‐5‐pyrazolone (DMPO) was purchased from Dojindo Ltd. KO_2_ and 18‐crow‐6 were purchased from Shanghai medical instrument Ltd (Shanghai, China).

### 4.2 Synthesis of SPRC@MPDA‐RGD Nanoparticles

Preparation of MPDA‐RGD nanoparticles: The synthesis of MPDA NPs was optimized with modifications in the previously reported method [36]. 0.15 g of dopamine hydrochloride and 0.1 g of F127 were stirred together in a mixed solution of deionized water and ethanol for 3 h. Then, 160 µL of TMB was added. Subsequently, the mixture was sonicated for 2 min, and 400 µL of ammonia solution was added. The mixture was then stirred for 2 h, after which it was centrifuged at 15,000 rpm and washed repeatedly with ethanol and water to yield the MPDA nanoparticles. c(RGDyK) was subsequently modified onto the MPDA surface via Michael addition reaction. Briefly, c(RGDyK) solution (2.5 mg/mL, 4.0 mL) was added into MPDA solution (2 mg/mL, 5 mL) and stirred for 24 h. The product was obtained via centrifugation (15000 rpm, 10 min) and washed with deionized water for three times.

Preparation of SPRC@MPDA‐RGD NPs: For SPRC loading, 1 mL different concentration of SPRC (1, 2, 4, 6, and 8 mg/mL) was pre‐dissolved in methanol, and then MPDA‐RGD (10 mg) was added in the SPRC solution and sonicated for 1 h. Afterwards, the resulting mixture was stirred for 24 h. The product was dialyzed for 3 days against 500 mL of deionized water (MW cut‐off: 3500 Da) and collected for further use. The preparation method of SPRC@MPDA is also the same as the above method. The SPRC loading capacity and encapsulation efficiency of SPRC@MPDA‐RGD were determined by HPLC method [73, 74].

### 4.3 Characterization

The morphologies of MPDA NPs were investigated by transmission electron microscopy (TEM). The TEM images and element mapping were captured using a JEOL microscope (JEM‐2100F, JEOL, Japan) at an accelerating voltage of 200 kV. While the hydrodynamic diameters and zeta potentials of MPDA NPs were measured by DLS (Zetasizer Ultral, Malvern Instruments, UK). Detection of UV‐visible absorption spectra was performed utilizing an enzyme labeling instrument (Tecan InfiniteM1000 Pro. Switzerland). The detection of free radicals was measured using an electron spin resonance spectrometer (ESR 5000, Bruker Magnettech, Germany).

### 4.4 Evaluation of Antioxidant Properties of MPDA, SPRC@MPDA, and SPRC@MPDA‐RGD

First, the Hydrogen Peroxide Kit (Solabio Ltd, Beijing, China) was used to evaluate the ability of MPDA, SPRC@MPDA, and SPRC@MPDA‐RGD to scavenge H_2_O_2_ as a means of validating peroxidase‐like activity. Hydrogen peroxide is able to bind titanium ions to form a yellow complex, and its absorbance at 415 nm provides feedback on the amount of hydrogen peroxide remaining, as a side‐by‐side assessment of the ability to scavenge hydrogen peroxide.

Secondly, the ·OH scavenging ability of MPDA, SPRC@MPDA, and SPRC@MPDA‐RGD was evaluated by EPR spectroscopy, employing DMPO as a trapping agent. Typically, DMPO (5 µL) was dissolved in PBS buffer (65 µL 10 mm), and then Fe^2+^ (10 µL, 50 mm) was added to catalyze the production of hydroxyl radicals (·OH) from H_2_O_2_. The amount of ·OH was determined by the intensity of the EPR amplitude with the addition of MPDA, SPRC@MPDA, and SPRC@MPDA‐RGD (80 µL, 200 µg/mL).

Finally, the ·O_2_^−^ scavenging capacity was also examined using EPR spectroscopy (Bruker MagnetTech ESR5000). 20 µg KO_2_ was added to 230 µL of DMSO solution containing 18‐crown ether‐6 (0.5 mm) to generate ·O_2_^−^, which was then further trapped by adding DMPO (20 µL). EPR signals were then collected with different MPDA, SPRC@MPDA, and SPRC@MPDA‐RGD (50 µL, 600 µg/mL) and quantitatively estimated to verify the ·O_2_^−^ scavenging ability.

### 4.5 Cell Culture

The mouse brain microvascular endothelial cell line (bEnd.3, RRIDs: CVCL_0170) was obtained from the American Type Culture Collection (ATCC, Manassas, VA, USA). Authentication of bEnd.3 cells was validated by short tandem repeat profiling.

10% foetal bovine serum (FBS) was added into Dulbecco's modified Eagle's medium (DMEM) for culture. And the cells were cultured in cell incubator (37°C; 5% CO_2_). As for the establishment of Oxygen‐glucose deprivation (OGD) model, the bEnd.3 cells were cultured with glucose‐deprived DMEM and placed in a hypoxic (<0.5% O_2_) incubator for 8 h to simulate the ischemic and hypoxic conditions following SCI.

### 4.6 Western Blot Analysis

The sample preparation and experimental methods of Western Blot are as previously described [60]. The primary antibodies’ information was shown in Table S1.

### 4.7 Immunofluorescence Staining

Following being fixed in 4% paraformaldehyde, tissue and cell samples were subjected to subsequent processing. First, samples were blocked with 5% BSA sufficiently. Subsequently, they were transferred to 4°C overnight with the specific primary antibody. After incubation, the samples were then incubated with the secondary antibody for 1 h. And at the end of the process, DAPI was added into the samples. The image was then documented using fluorescence microscope (BZ‐X800E, Keyence, Japan) and measured by ImageJ software. The primary antibodies used were listed in Table S1.

### 4.8 ROS Assay

After different interventions, the cells were co‐incubated with DCFH‐DA for 30 min. The nuclei were subsequently stained with the Hoechst dye. Finally, images were documented by fluorescence microscope (BZ‐X800E, Keyence, Japan). In addition, the stained cells with different treatments were also tested and analyzed for ROS levels by a flow cytometer (Sysmex Partec, Japan).

To detect the oxidative stress in spinal cord tissue, the fresh spinal cord was removed and prepared into frozen sections. Then, a 30‐min incubation of the sections with DHE dye at 37°C was performed in the dark. Finally, the sections were documented as previously described.

### 4.9 Trans‐endothelial Permeability Assay In Vitro

The measurement of trans‐endothelial permeability was conducted in vitro, as reported previously [60]. Briefly, bEnd.3 cells were seeded into transwell chamber until reaching confluence, and then OGD was conducted.FITC‐dextran transport from the upper to the lower chamber was detected by measuring fluorescence intensity with a microplate reader.

### 4.10 Animals

The eight‐week‐old C57BL/6 mice were purchased from SPF (Suzhou) Biotechnology Co., Ltd. (Jiangsu, China) and maintained under standard conditions with a 12‐h light–dark cycle and controlled temperature (22.5±2.5°C) and humidity (60 ± 10%). All food and water are available without restriction. All animal experiments were conducted in accordance with the Guide for the Care and Use of Laboratory Animals from the National Institutes of Health. All the experimental procedure were approved by the Institutional Animal Care and Use Committee of Shanghai Shengchang Biotechnology Co., Ltd. (No: 2024‐01‐LY‐LXF‐110).

### 4.11 Induction of Contusive SCI Model and Experimental Design

Prior to surgery, isoflurane (RWD life science, China) was used to anaesthetize all mice. The skin of the surgical site was prepared and disinfected after anesthesia induction and positioning. Laminectomy was then performed at T10 vertebrae. Subsequent to T10 laminectomy, a spinal cord contusion was delivered at the T10 spinal cord by RWD's Precision Strike. The detailed experimental parameters employed in the contusive SCI model were set as reported before [75]. The mice in sham group only received the laminectomy but without injury induction. After suturing the wound, the mice were transferred to a warm room. During the experimental period, the mice were subjected to manual urination twice daily.

After the baseline motor function was evaluated using the BMS score, mice were randomly assigned to different groups. Mice in the treatment group received MPDA, SPRC, SPRC@MPDA, SPRC@MPDA‐RGD by tail vein injection accordingly. The same dose of SPRC (20 mg/kg) was used by the SPRC, SCI+SPRC@MPDA, and SCI+SPRC@MPDA‐RGD groups. The initial treatment was administered immediately following SCI, followed by further treatments on days 1, 3, and 5 after the injury. Spinal cord samples were then collected on the seventh day after injury for subsequent experiments.

### 4.12 Determination of SPRC@MPDA‐RGD Uptake In Vivo

First, the fluorescently labeled drug was prepared. Typically, SPRC@MPDA/SPRC@MPDA‐RGD and NHS‐Cy5.5 are mixed at a feeding ratio of 10:1, stirred overnight, then centrifuged for purification, and finally diluted with physiological saline to the required concentration for later use. After depilating the back, isoflurane was used to anaesthetize. After injecting different groups of drugs, a small animal in vivo imaging instrument was used to evaluate the targeting ability.

### 4.13 Behavioral Assessment

The inclined plane test and the Basso mouse scale (BMS) behavioral assessment were performed to evaluate motor function recovery following injury. Briefly, the BMS scores were executed by two researchers blinded to the group assignments to assess the hind‐limb motility function in mice. The observations were evaluated by placing the mice in an open field that had been acclimatized before. Mice were put on a flat plate with increasing angles in turn for the inclined plane test, and the maximum angle they could remain stationary for more than 5 s was recorded.

### 4.14 Electrophysiological Assay in Mice

At 28 days after SCI, electrophysiological assay was conducted to assess motor evoked potentials (MEP) in mice. After anaesthetization, the mice were fitted with stimulating electrodes that were implanted intracranially from behind the ear, while recording electrodes were placed in the gastrocnemius muscle to capture MEP.

### 4.15 HE Staining and Nissl Staining

After sampling, spinal cords from different groups were fixed in 4% paraformaldehyde on day 7 post SCI. After fixation, the samples were removed out for pathological sectioning. Rinsing with distilled water, staining with hematoxylin solution, the sections were subsequently subjected to dehydration by using alcohol and then continued to stain with eosin dye for 3 min. As for Nissl staining, the prepared sections were first rinsed with distilled water and subsequently incubated with cresyl violet for 20 min.

### 4.16 Mouse Inflammatory Cytokines Assay

The spinal cords of mice were sampled, subsequently lysed with RIPA buffer, and centrifuged to obtain the supernatant. The levels of inflammatory cytokines were then determined by using the RayPlex^TM^ Mouse Inflammation Array 1 kit (RayBiotech, Guangzhou, China) following the manufacturer's guidelines.

### 4.17 BSCB Permeability Assessment

The BSCB permeability was detected by Evans blue (EB, MedChemexpress, USA) dye extravasation method. Briefly, on day 7 after modelling of SCI, mice were intravenously injected with 2% Evans Blue and waited for the dye to circulate for 3 h. Subsequently, the mice were then anesthetized and euthanized. The injury site of the spinal cords was then extracted, imaged, and weighed for qualitative measurement. The injury lesions of the spinal cord were then co‐incubated with N, N‐dimethyl formamide at 72°C for 3 days. After collecting supernatants, the optical density was quantified with a microplate reader at 620 nm excitation/680 nm emission to analyze the EB dye content.

### 4.18 RNA Extraction, Bulk RNA Sequencing, and Data Analysis

The samples of spinal cord were collected from mice in SCI, SCI+SPRC@MPDA‐RGD, and Sham group respectively. RNA from the samples was isolated and purified using TRIzol (Thermo Fisher, USA) according to the manufacturer's protocol. The amount and purity of total RNA were then controlled using NanoDrop ND‐1000 (NanoDrop, Wilmington, DE, USA), and the integrity of RNA was tested using Bioanalyzer 2100 (Agilent, CA, USA); concentration >50 ng/µL, RIN value >7.0, and total RNA >1 µg were sufficient for downstream experiments. Oligo(dT) magnetic beads (Dynabeads Oligo (dT), Thermo Fisher, USA) were used for two rounds of purification to specifically capture mRNA with PolyA. The captured mRNA was fragmented under high temperature conditions using a magnesium ion fragmentation kit (NEBNext^R^ Magnesium RNA Fragmentation Module, New England Biolabs, USA) at 94°C for 5–7 min. The fragmented RNA was synthesized into cDNA by reverse transcriptase (Invitrogen SuperScript^TM^ II Reverse Transcriptase, CA, USA). Then, E. coli DNA polymerase I (New England Biolabs, USA) and RNase H (New England Biolabs, USA) were used for double‐strand synthesis to convert the DNA and RNA composite double strands into DNA double strands. At the same time, dUTP Solution (Thermo Fisher, USA) was incorporated into the second strand to fill the ends of the double‐stranded DNA into blunt ends. Then, an A base was added to each end to enable it to be connected to the adapter with a T base at the end, and the fragment size was screened and purified using magnetic beads. The second strand was digested with UDG enzyme (New England Biolabs, USA), and then PCR was performed‐pre‐denaturation at 95°C for 3 min, denaturation at 98°C for a total of 8 cycles for 15 s each, annealing at 60°C for 15 s, extension at 72°C for 30 s, and finally extension at 72°C for 5 min to form a library with a fragment size of 300 bp ± 50 bp (strand‐specific library). Finally, we used Illumina NovaseqTM 6000 (LC Bio Technology CO., Ltd. Hangzhou, China) for double‐end sequencing according to standard operations, and the sequencing mode was PE150.

The FastQC v0.10.1 was used to assess the quality of fastq files. Reads were aligned by Hisat2 2.0 to the mm10 version (Dec 2019) of the mouse genome [76]. The mapped reads were assembled using StringTie. The transcriptomes were merged to reconstruct a comprehensive transcriptome. StringTie and ballgown were used to estimate the expression levels of all transcripts and perform expression abundance for mRNAs by calculating FPKM value [77]. Principal component analysis was used to identify outliers, and then differential expression was performed using R package edgeR [78]. Genes with false discovery rate (FDR) calculated by *p*‐value adjusted for Benjamini‐Hochberg < 0.05 and absolute log2FC > 0.585 were considered differentially expressed.

### 4.19 Measurement of Fe^2+^ Content

Ferrous Iron Colorimetric Assay Kit (Elabscience, China) was utilized for detecting Fe^2+^ concentration. Subsequently, the corresponding absorbance at 593 nm was detected. To detect the Fe^2+^ content in the spinal cord, supernatants were also prepared using the manufacturer's protocol and measured as previously described.

### 4.20 Measurement of Oxidative Stress

The Lipid Peroxidation MDA Assay Kit and the Total Superoxide Dismutase Assay Kit with WST‐8 obtained from Beyotime Institute of Biotechnology were used following the manufacturer's guidelines to determine the level of superoxide dismutase (SOD) and malondialdehyde (MDA) in bEnd.3 cells subjected to different interventions. To detect the level of oxidative stress of tissues, the spinal cord samples were collected from different groups, and then the same procedure was also performed to calculate the level of MDA or SOD.

### 4.21 Measurement of H_2_S Concentration

The Endogenous H_2_S assay kit (Nanjing Jiancheng Bioengineering Institute, China) was utilized to assess the H_2_S level following the manufacturer's guideline. After sample preparation, the absorbance of methylene blue produced from the assay kit produced by H_2_S was quantified at 665 nm to determine the H_2_S concentration in each group.

### 4.22 Biosafety Evaluation

In separate experiment, C57BL/6 mice were only administered with SPRC@MPDA‐RGD individually. Then, mice were subsequently anaesthetized and euthanized on days 1, 3, 7, 14, and 28. Major organs were isolated for HE staining and blood samples were collected for serological examinations to comprehensively assess the biosafety of SPRC@MPDA‐RGD in vivo.

### 4.23 Statistical Analysis

All statistical assays were conducted by using GraphPad Prism 9.5. All the data were subjected to the assessment of normality and equality of variances prior to conducting statistical comparisons. Unless otherwise stated, parametric data were shown as mean ± standard deviation (SD). The sample size of the statistical analysis is documented in the figure legends separately. Statistical comparisons between two groups were analyzed using two‐tailed, unpaired Student's t‐test. And comparisons among multiple groups were analyzed by one‐way or two‐way ANOVA in conjunction with the Tukey's multiple comparisons tests, with a *p* value<0.05 considered statistically significant.

## Author Contributions

Z.C., X.P., R.L., and Y.W. contributed equally to this work. Z.C. and X.P. conceptualized the study. Z.C., X.P., R.L., and Y.W. developed the methodology. Z.C., X.P., Q.Z., J.W., and Y.Q. conducted the investigation. Z.C., X.P., and F.L. created the visualization. J.Z., Z.S., J.S., and X.L. supervised the project. Z.C., X.P., R.L., and Y.W. wrote the original draft. J.Z., Z.S., J.S., and X.L. reviewed and edited the manuscript.

## Funding

The National Key Research and Development Program of China (2024YFC2419600); the National Natural Science Foundation of China (82572758); the Natural Science Foundation of Shanghai (23ZR1448100), and the Open Research Fund of Naval Medical University Basic Medical College (ORFBMC‐JCKFKT‐MS‐012).

## Conflicts of Interest

The authors declare no conflicts of interest.

## Supporting information




**Supporting File**: advs74716‐sup‐0001‐SuppMat.docx

**Supporting File**: advs74716‐sup‐0002‐Data.pdf

## Data Availability

The data that support the findings of this study are available from the corresponding author upon reasonable request.
