# Supplementary material for: Targeted H2S Delivery System Attenuates Blood‐Spinal Cord Barrier Disruption after Spinal Cord Injury by Reshaping the Ferritinophagy Pathway
Source: Adv Sci (Weinh). 2026 Mar 10;13(28):e18901. doi: 10.1002/advs.202518901 (PMC13185822; doi:10.1002/advs.202518901)
Supplement: Supplementary file 1 — Supporting File: advs74716‐sup‐0001‐SuppMat.docx [file ADVS-13-e18901-s001.docx]

Supporting Information

**Targeted H_2_S Delivery System Attenuates Blood-Spinal Cord Barrier Disruption after Spinal Cord Injury by Reshaping the Ferritinophagy Pathway**

*Zhiheng Chen; Xinkai Pu; Ruiyang Li; Yuezhou Wu; Qirong Zhou; Jian Wang; Yuxuan Qian; Fengjie Lu;* *Jian-Yuan Zhao ^*^**; Zhida Su^*^; Jiacan Su^*^; Xiaofeng Lian^*^*

Zhiheng Chen, Xinkai Pu, Ruiyang Li and Yuezhou Wu contributed equally to this work.

**Supporting figures**

**Figure S1**

**

**

**Figure S1.** Scavenging H_2_O_2_ activity of SPRC@MPDA-RGD at different concentrations.

**Figure S2**


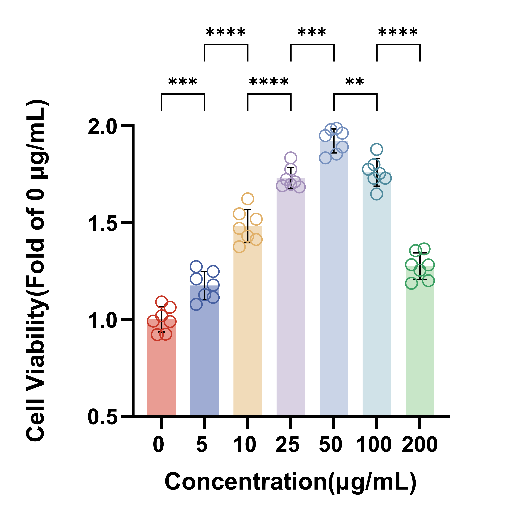


**Figure S2.** CCK-8 detected the viability of bEnd.3 after OGD in different concentrations of SPRC@MPDA-RGD. Data are expressed as mean ± SD (n = 7). ** p < 0.01, *** p < 0.001, **** p < 0.0001, one-way ANOVA with Tukey's multiple comparisons tests.

**Figure S3**

**
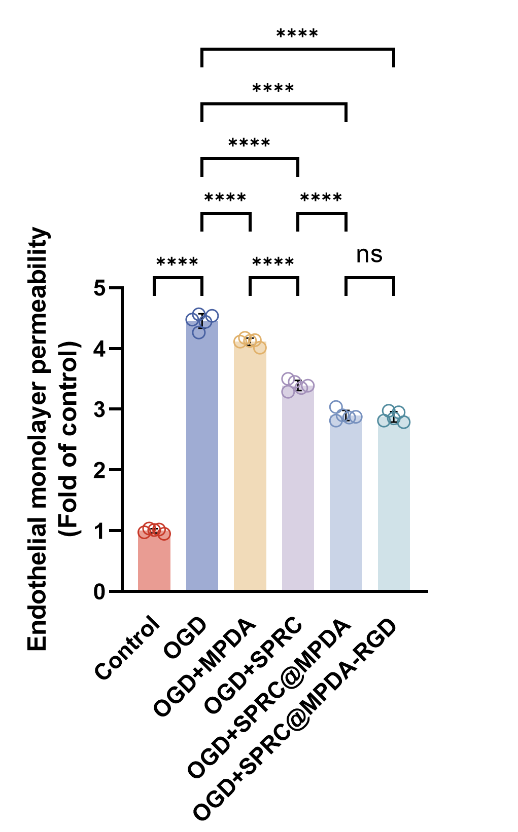
**

**Figure S3.** The trans-endothelial permeability assay was utilized to assess the permeability of bEnd.3 cells after different treatments. Data are expressed as mean ± SD (n = 5). **** p < 0.0001, ns means no significance, one-way ANOVA with Tukey's multiple comparisons tests.

**Figure S4**

**
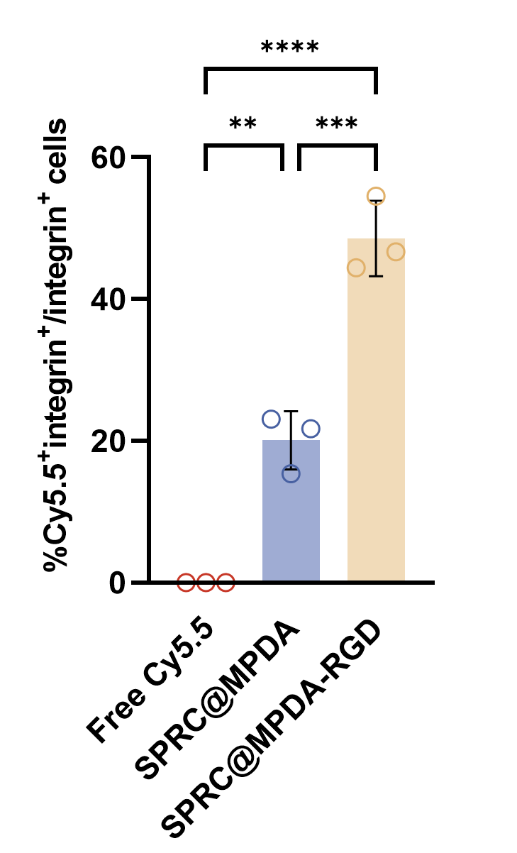
**

**Figure S4.** Quantification of the proportion of Cy5.5^+^integrin^+^ cells among total integrin^+^ cells in different groups. Data are expressed as mean ± SD (n = 3). ** p < 0.01, *** p < 0.001, **** p < 0.0001, one-way ANOVA with Tukey's multiple comparisons tests.

**Figure S5**

**
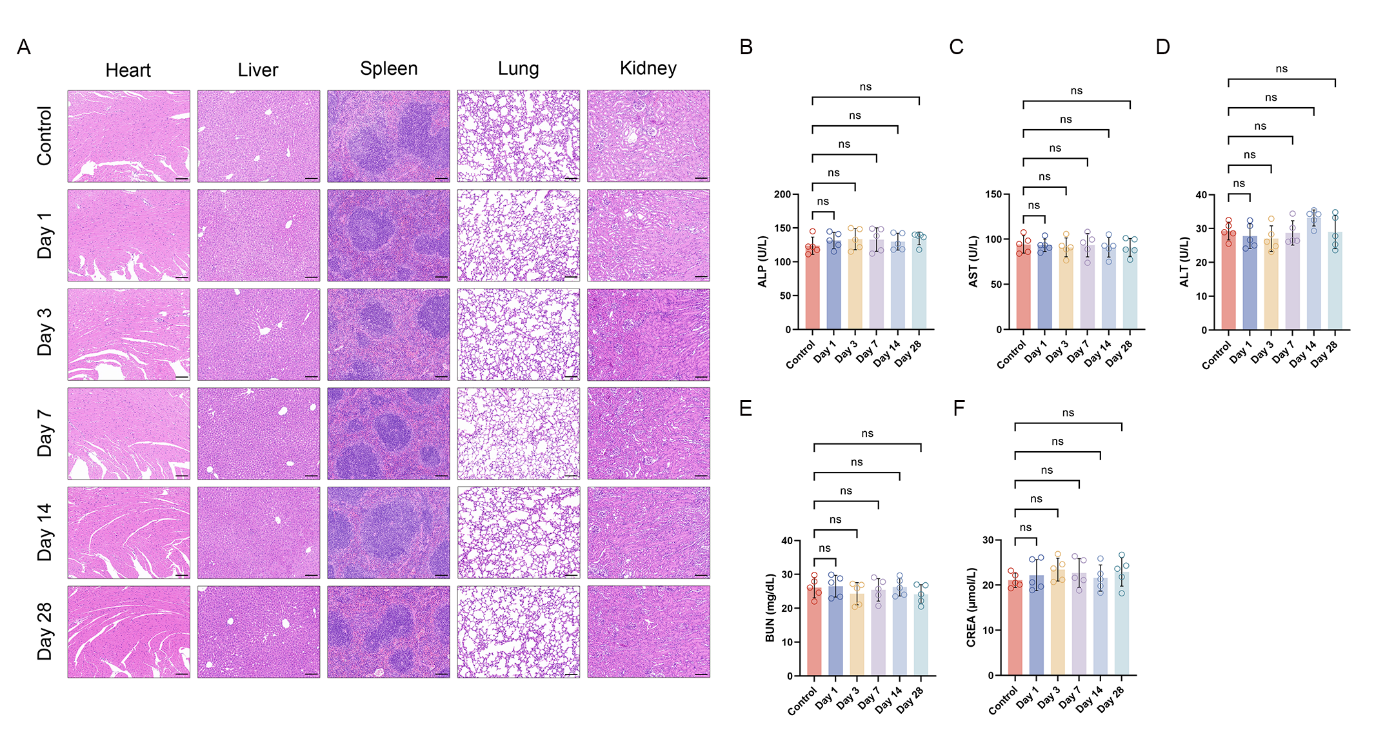
**

**Figure S5.** A) The main organs’ representative HE staining of the mice; scale bar: 100 μm. B-F) The results of blood analysis parameters such as ALP, AST, ALT, BUN and CREA of the mice. Data are expressed as mean ± SD (n = 5). ns means no significance.

**Figure S6**

**
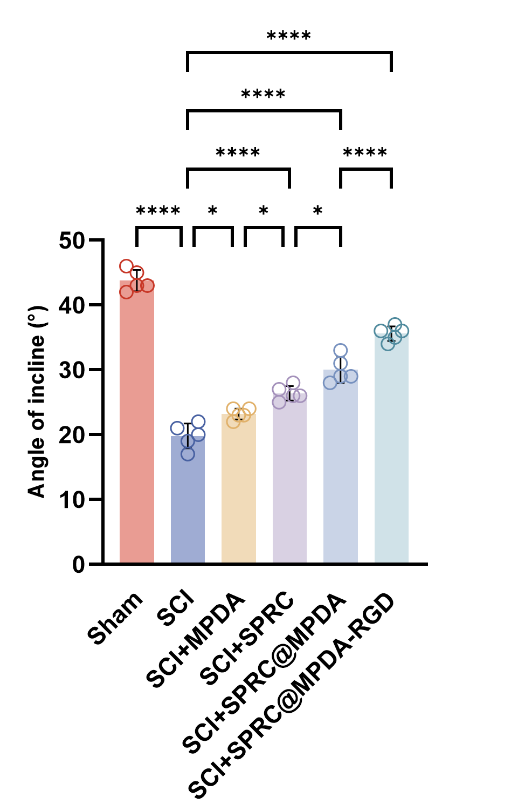
**

**Figure S6.** The inclined plane test was utilised to evaluate the motor function recovery in different groups of mice on the 14th day after SCI. Data are expressed as mean ± SD (n = 5). * p < 0.05, **** p < 0.0001, one-way ANOVA with Tukey's multiple comparisons tests.

**Figure S7**

**
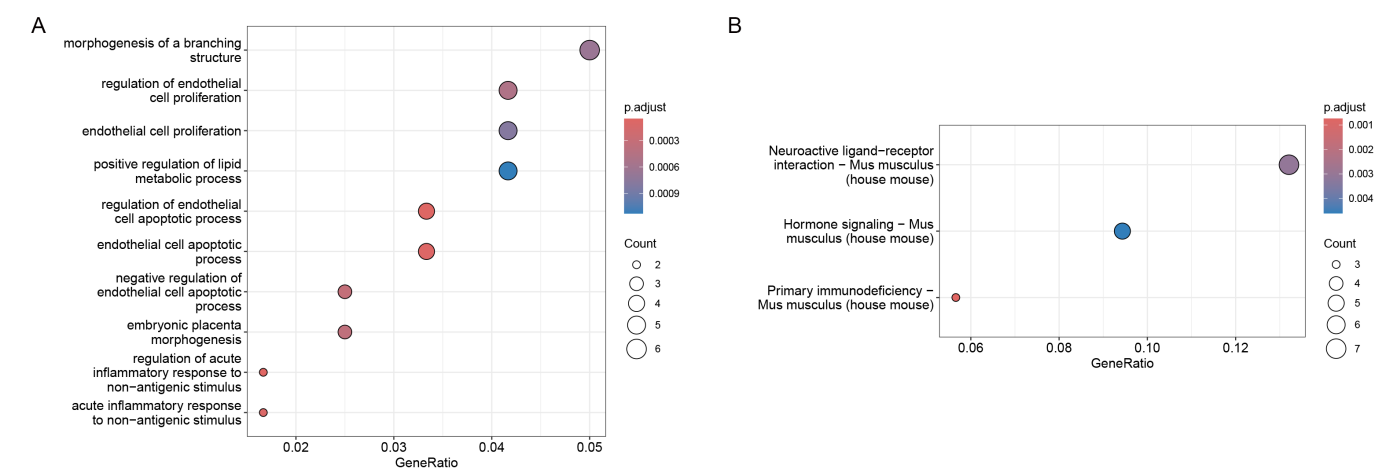
**

**Figure S7.** A) The result of GO enrichment analysis on 167 genes uniquely altered in the comparison between SCI group and SCI+SPRC@MPDA-RGD group. B) The result of KEGG pathway analysis on 167 genes uniquely altered in the comparison between SCI group and SCI+SPRC@MPDA-RGD group.

**Figure S8**


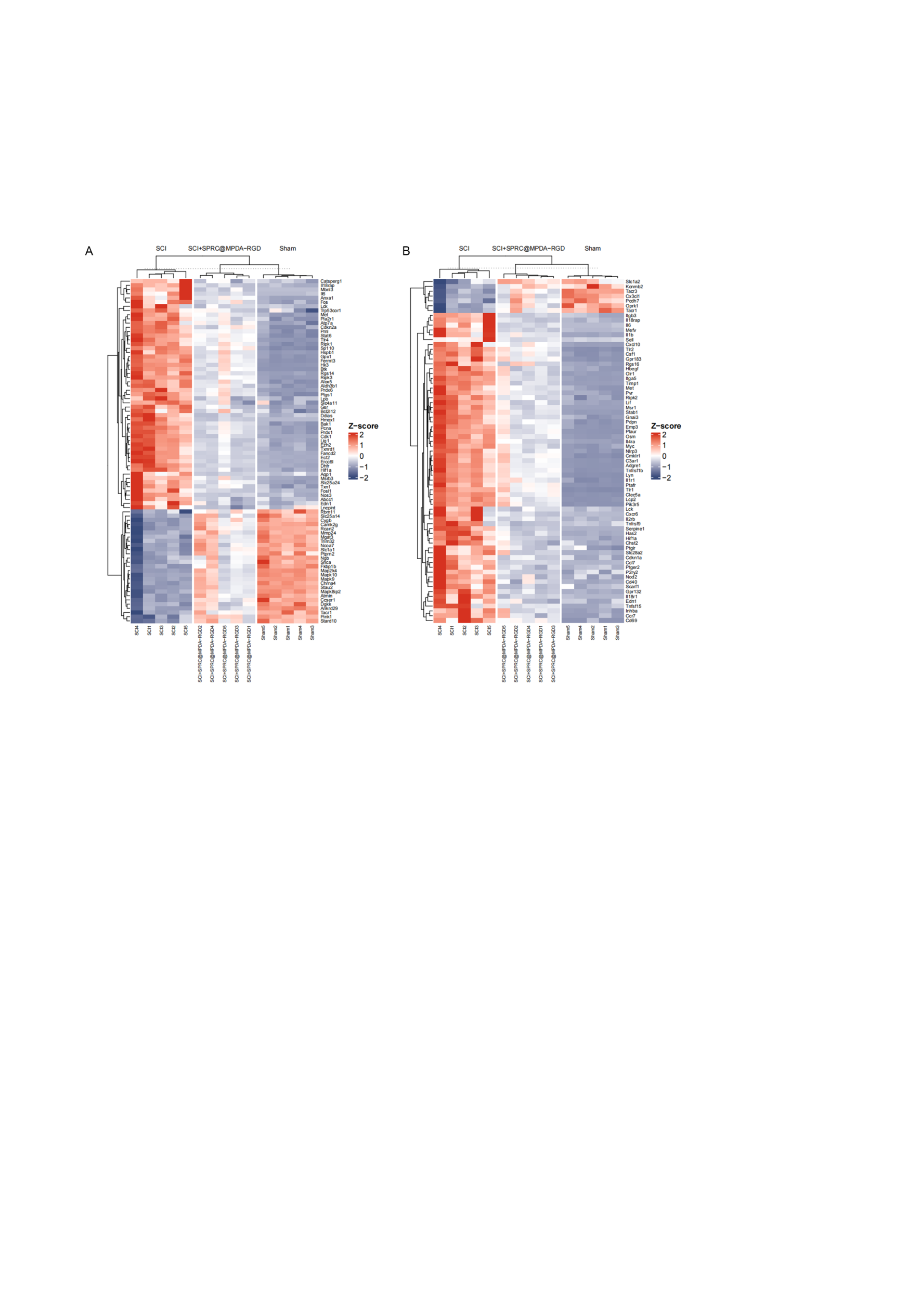


**Figure S8.** A-B) RNA-seq revealed differential gene expression and pathway modulation after treatment in mice after SCI, SCI+SPRC@MPDA-RGD, and Sham. Heatmaps show differential genes expression related to antioxidant (A) and anti-inflammatory (B) pathways.

**Figure S9**


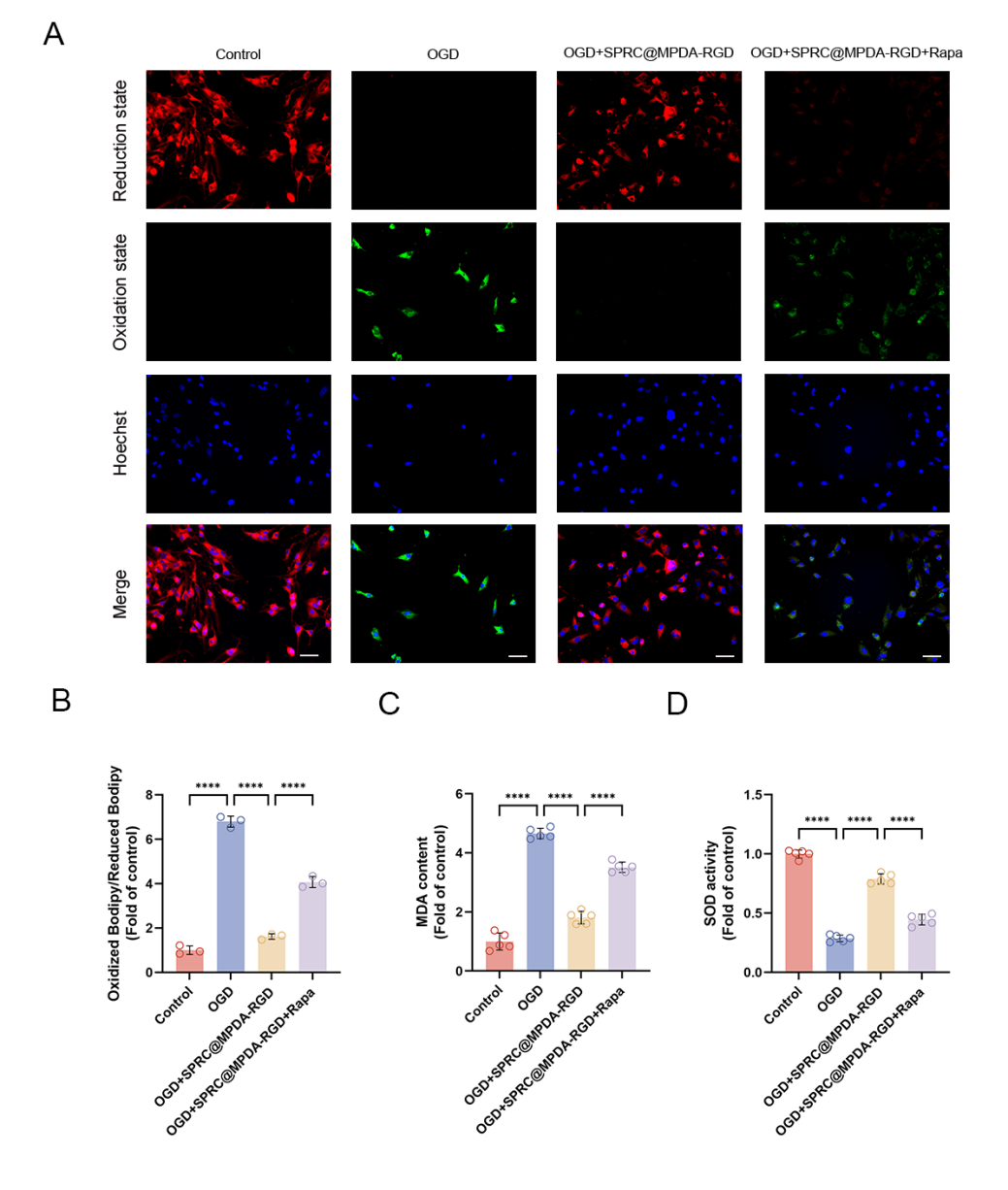


**Figure S9.** A, B) Representative images and quantitative analysis results of intracellular lipid ROS levels assessed by C11-BODIPY probe (n = 3). The fluorescence of the probe transitioned from red to green, indicating a significant increase of lipid peroxidation. Scale bar = 50 µm. C) MDA content levels in bEnd.3 cells (n = 5). D) SOD activity in bEnd.3 cells in different groups (n = 5). Data are expressed as mean ± SD. **** p < 0.0001, one-way ANOVA with Tukey's multiple comparisons tests.

**Table S1: Antibodies used for Western blotting (WB) and immunofluorescence (IF).**

| Antibody | Catalogue no | Company | Application |
| --- | --- | --- | --- |
| rabbit anti‐β-Catenin | 51067-2-AP | Proteintech | WB, IF |
| rabbit anti‐Occludin | 27260-1-AP | Proteintech | WB, IF |
| rabbit anti‐ZO-1 | 21773-1-AP | Proteintech | WB |
| rabbit anti‐Claudin-5 | #49564 | CST | WB |
| rabbit anti‐FTH | A19544 | ABclonal | WB |
| mouse anti‐FTL | 68068-1-Ig | Proteintech | WB |
| rabbit anti‐CSE | ab151769 | Abcam | WB |
| rabbit anti‐PI3K | A4992 | Abclonal | WB |
| rabbit anti‐p-PI3K | #4228 | CST | WB |
| rabbit anti‐Akt | 10176-2-AP | Proteintech | WB |
| rabbit anti‐p-Akt | T56569 | Abmart | WB |
| rabbit anti‐mTOR | #2983 | CST | WB |
| rabbit anti‐p-mTOR | #5536 | CST | WB |
| rabbit anti‐LC3 | #12741 | CST | WB, IF |
| rabbit anti‐NCOA4 | #DF4255 | Affinity | WB |
| rabbit anti‐GAPDH | 10494-1-AP | Proteintech | WB |
| mouse anti-Integrin | sc-7312 | Santa | IF |
