# Supplementary material for: Targeted H2S Delivery System Attenuates Blood‐Spinal Cord Barrier Disruption after Spinal Cord Injury by Reshaping the Ferritinophagy Pathway
Source: Adv Sci (Weinh). 2026 Mar 10;13(28):e18901. doi: 10.1002/advs.202518901 (PMC13185822; doi:10.1002/advs.202518901)

Uncropped Western blots

Figure 2E

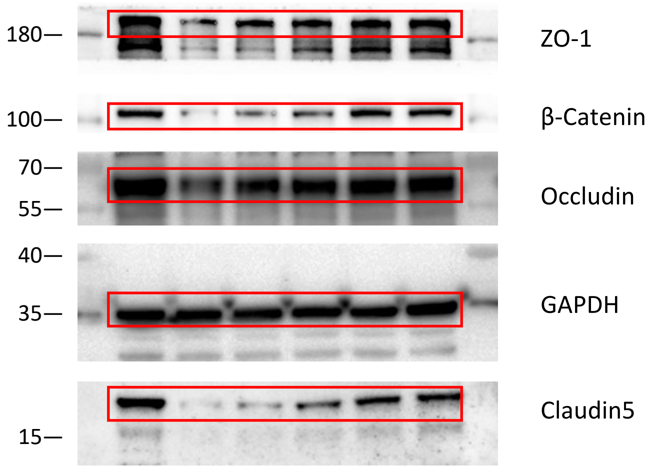

Figure 5C

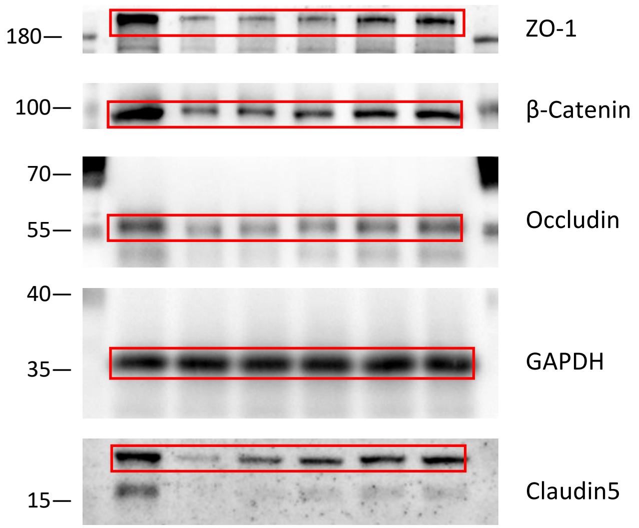

Figure 7I

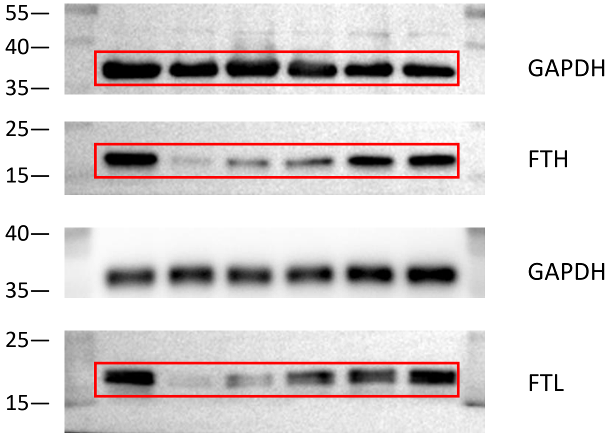

Figure 8B

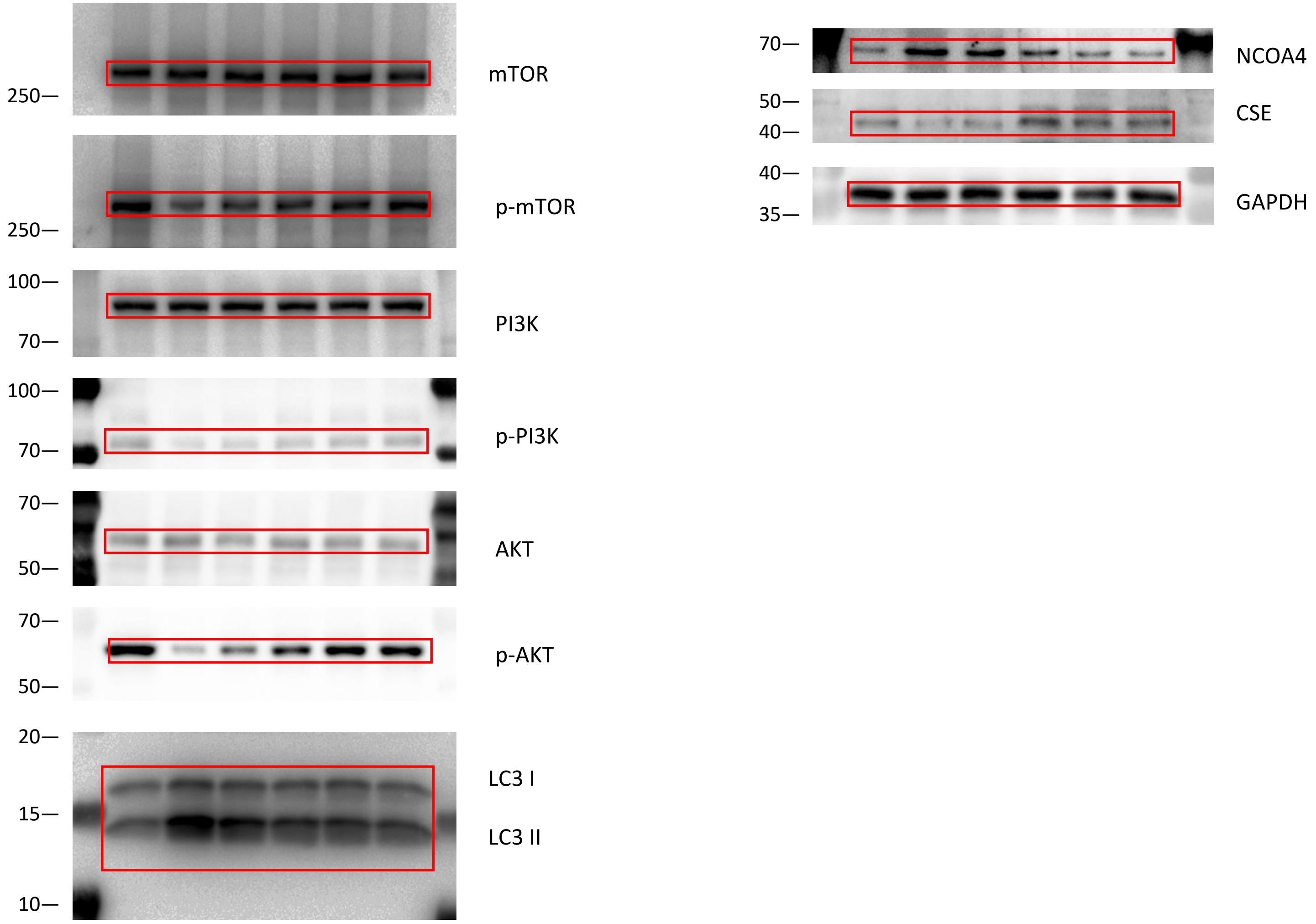
250—
mTOR
250—
p-mTOR
100—
70—
PI3K
100—
70—
p-PI3K
70—
50—
AKT
70—
50—
p-AKT
20—
15—
10—
LC3 I
LC3 II
70—
NCOA4
50—
40—
CSE
40—
35—
GAPDH

Microscope images

Figure 1A

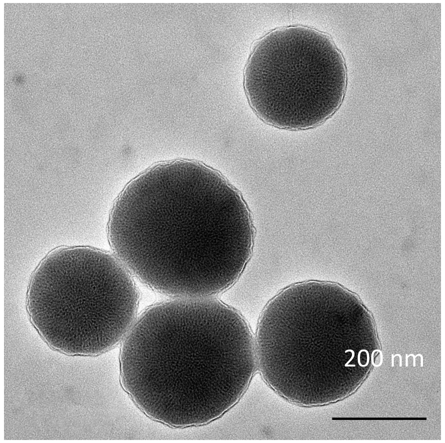

Figure 1B

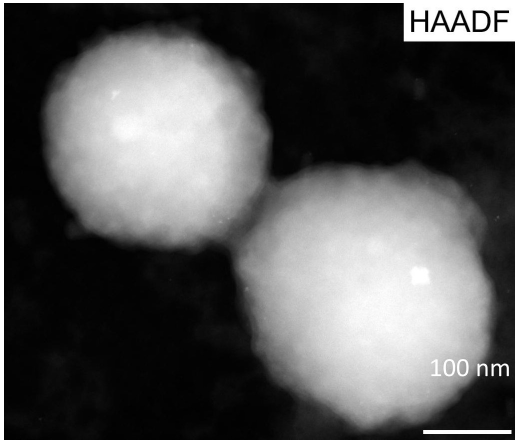

Figure 1C

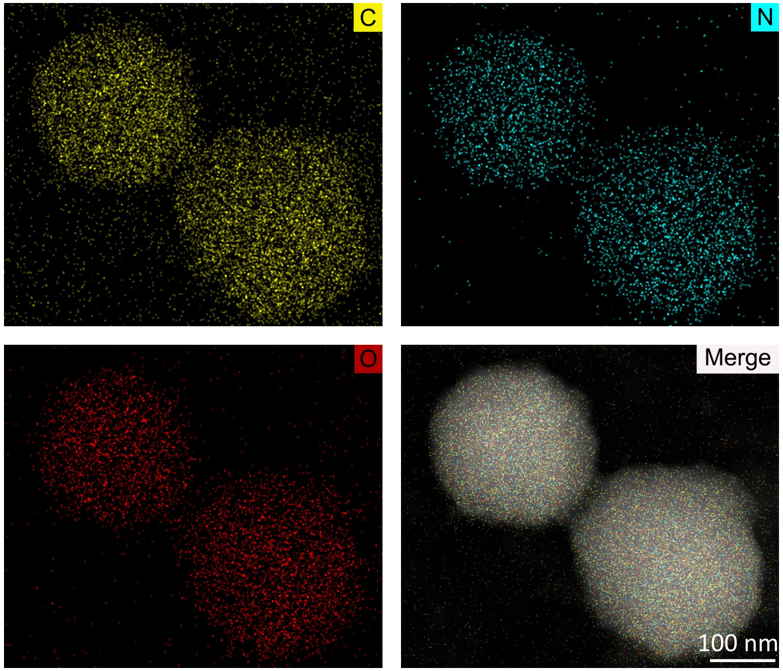

Figure 2B

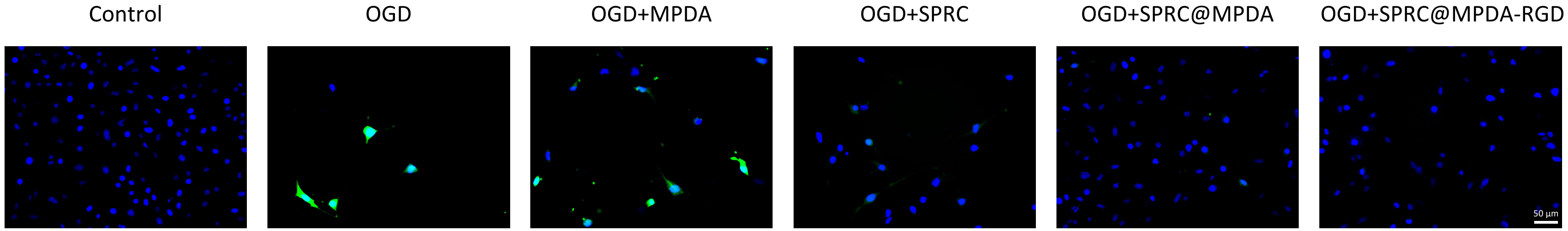

Figure 2G

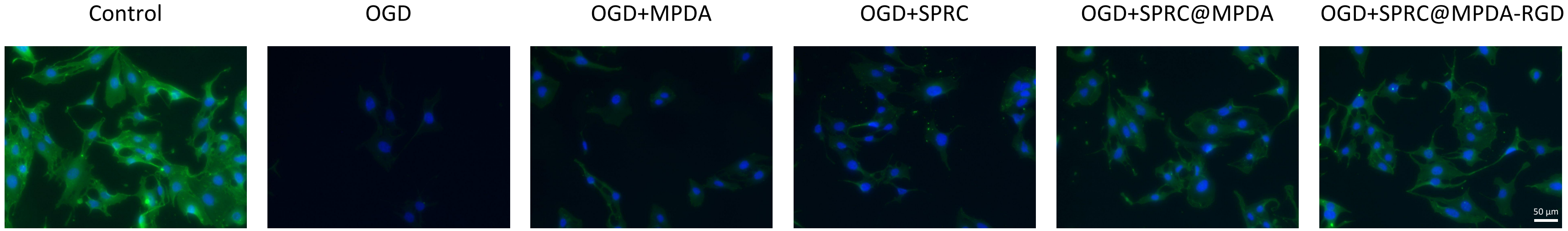

Figure 2I

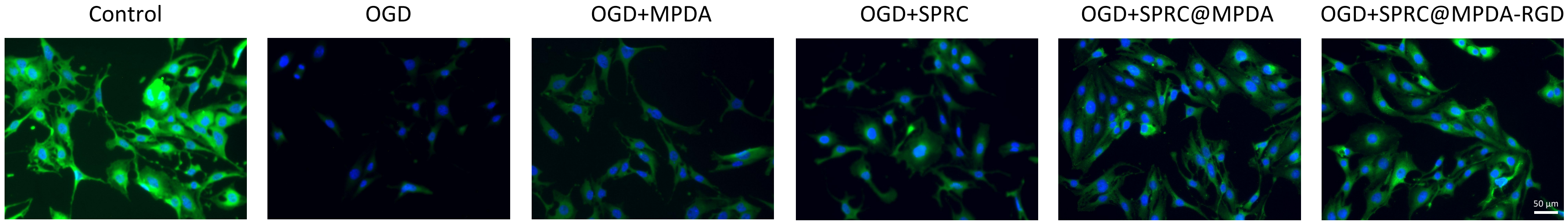

Figure 3E

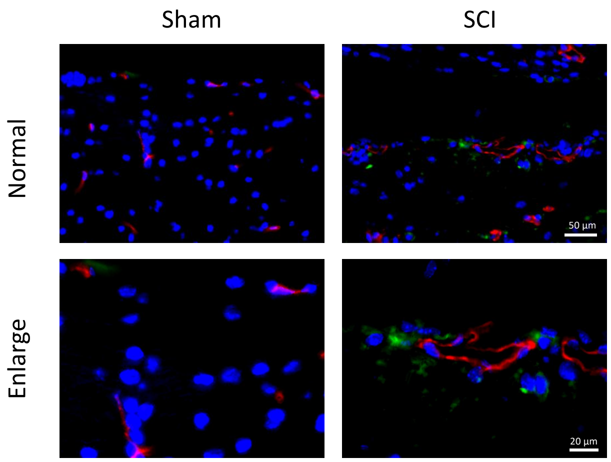

Figure 3G

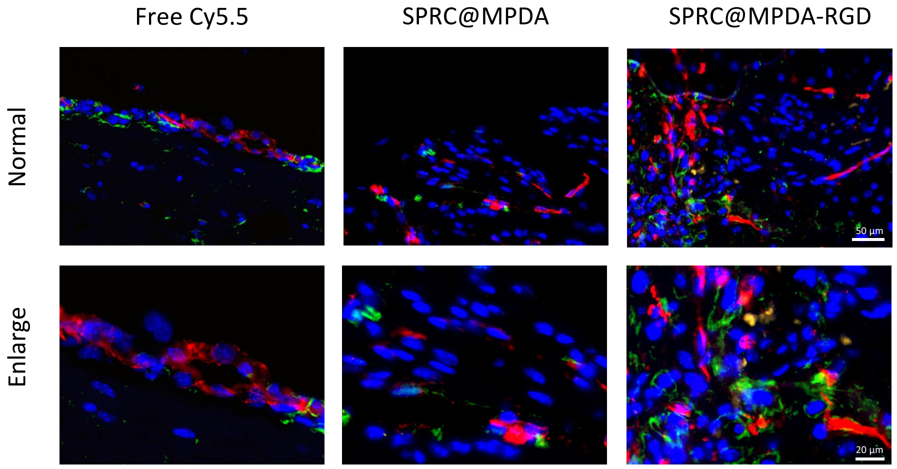

Figure 4C

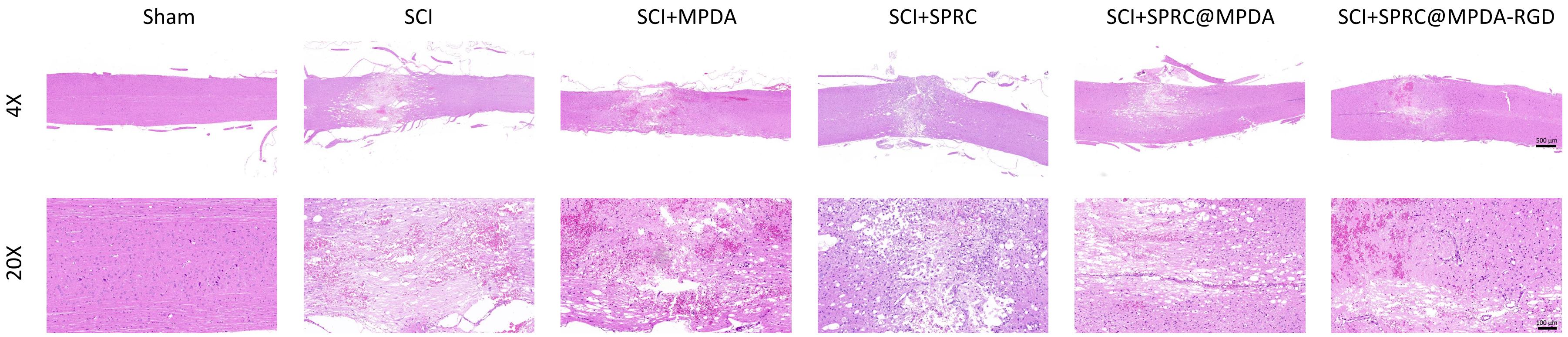

Figure 4D

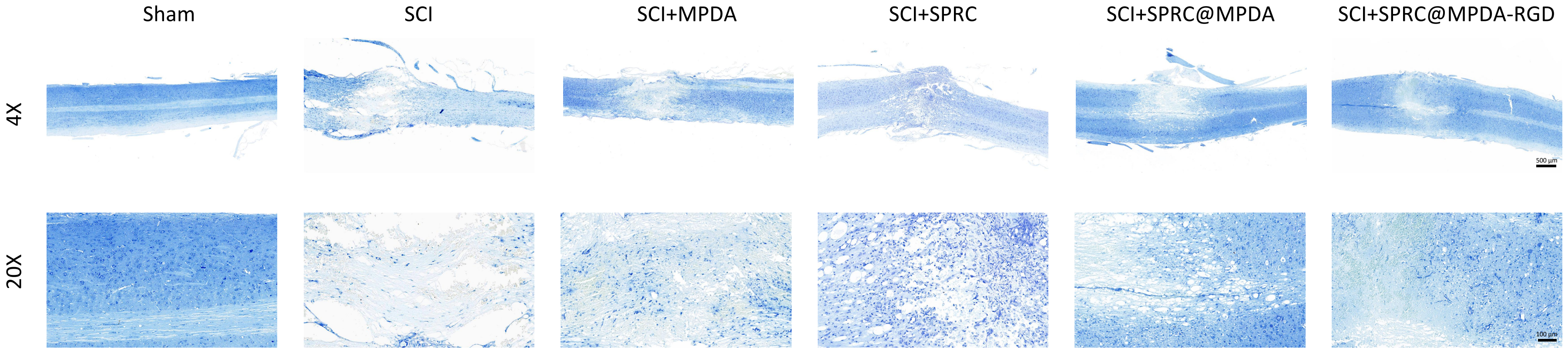

Figure 4F

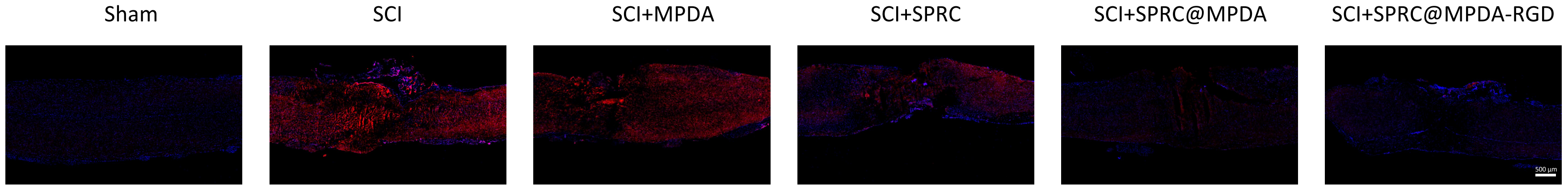

Figure 5F

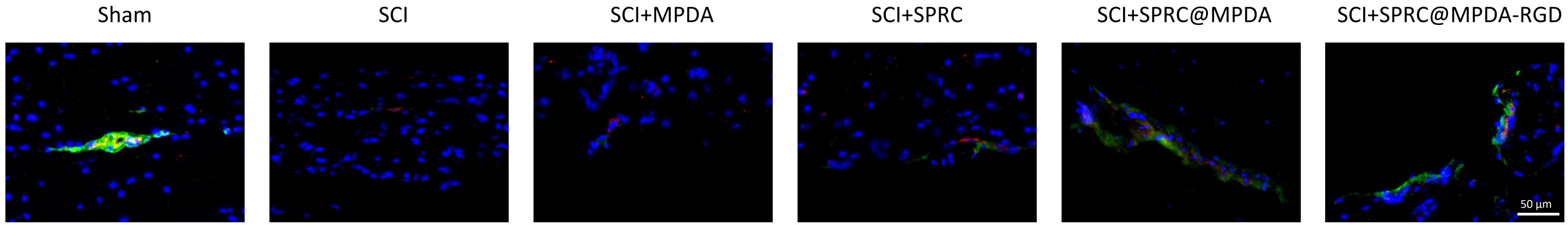

Figure 5H

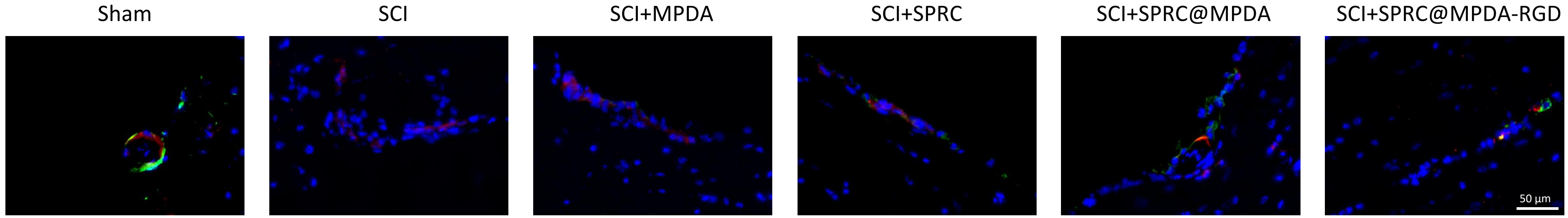

Figure 7B

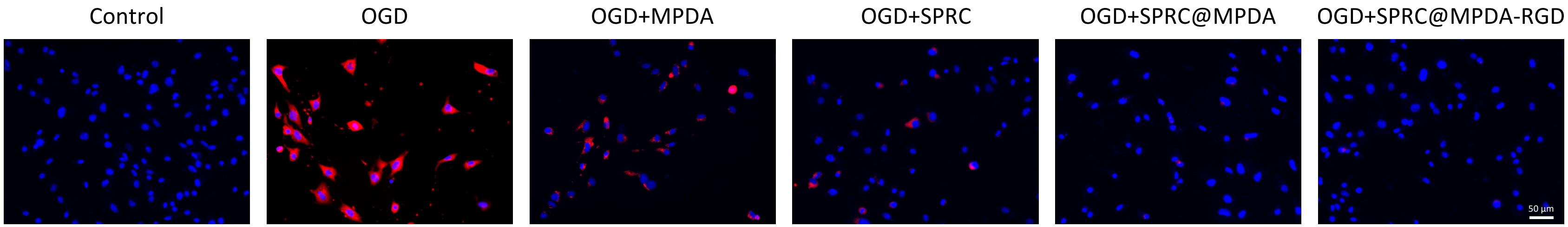

Figure 7E

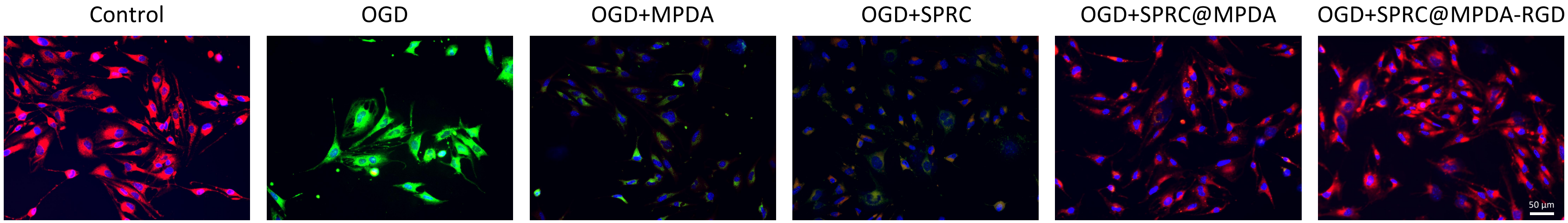

Figure 7L

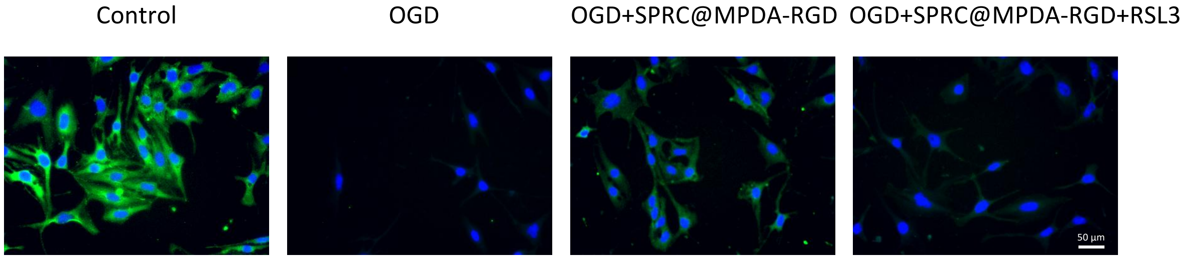

Figure 7N

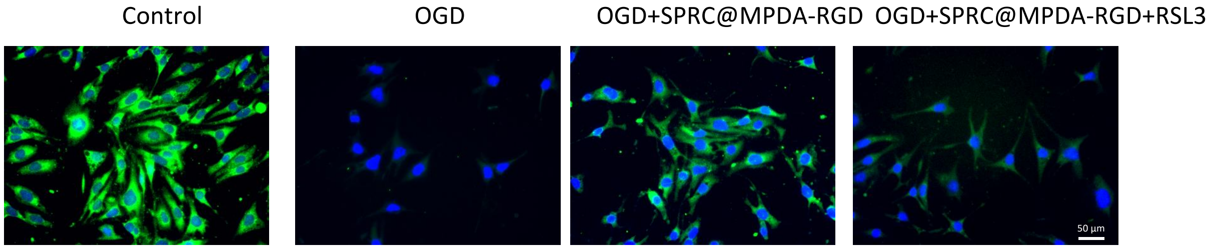

Figure 8E

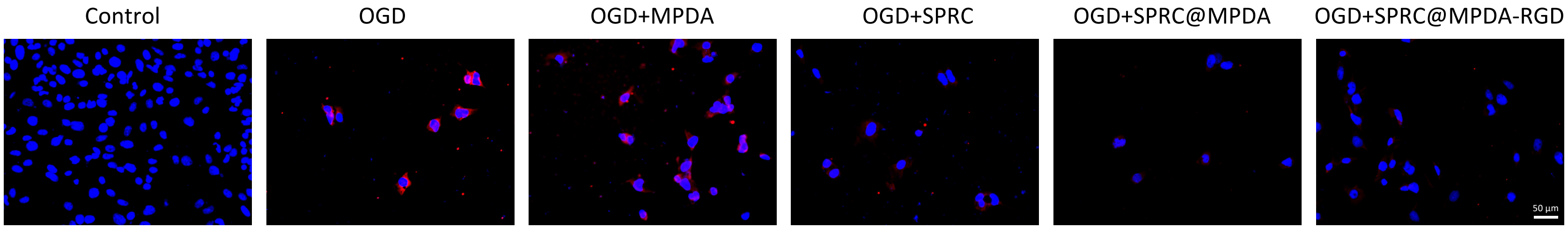

Figure 8G

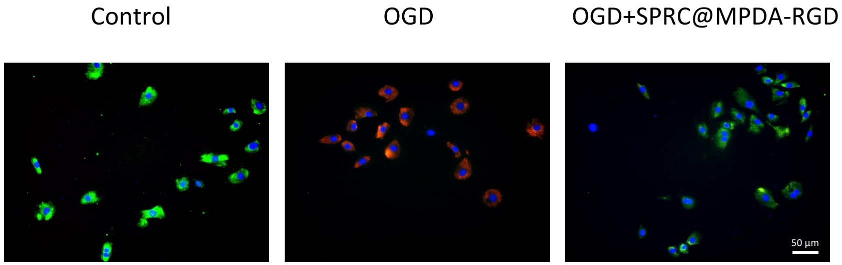

Figure 8H

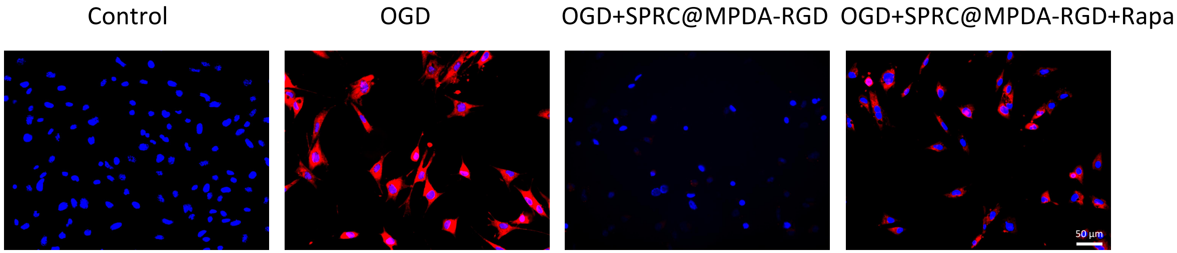

Figure 9D

Sham SCI SCI+MPDA SCI+SPRC SCI+SPRC@MPDA SCI+SPRC@MPDA-RGD

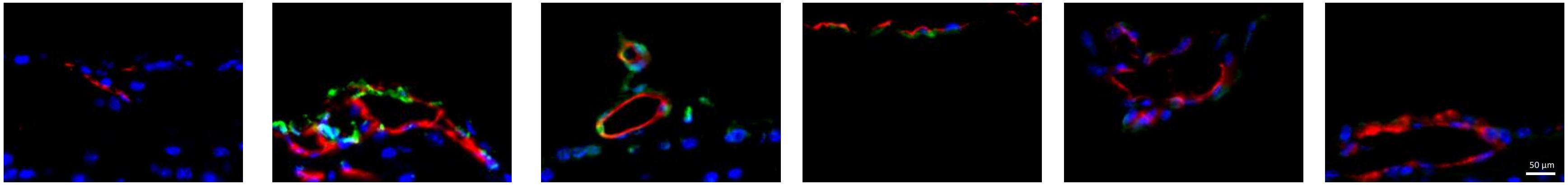

Figure 9F

Sham SCI SCI+MPDA SCI+SPRC SCI+SPRC@MPDA SCI+SPRC@MPDA-RGD

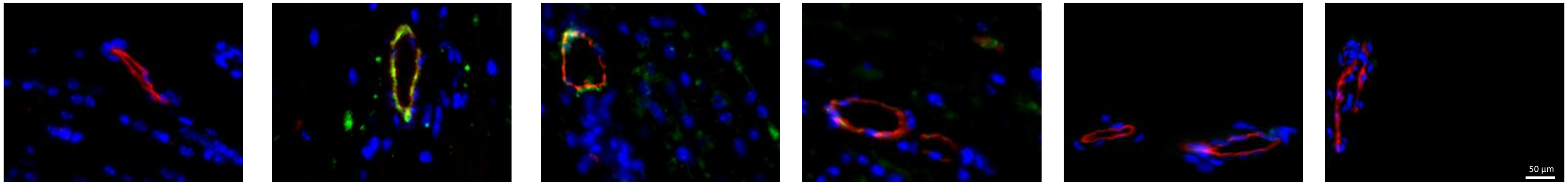

Figure S5A

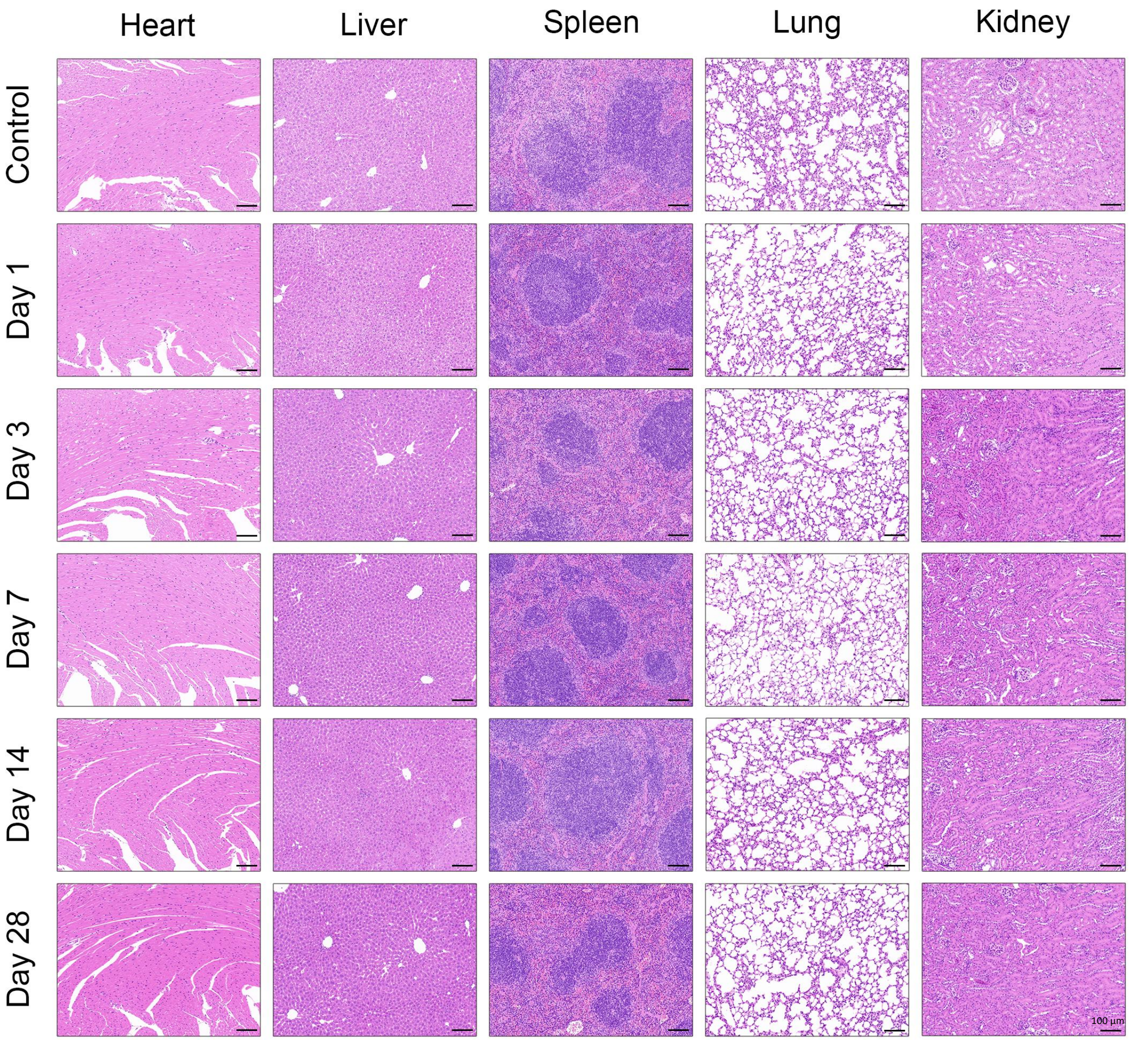
Heart
Liver
Spleen
Lung
Kidney
Control
Day 1
Day 3
Day 7
Day 14
Day 28
100 µm

Figure S9A

Control OGD OGD+SPRC@MPDA-RGD OGD+SPRC@MPDA-RGD+Rapa

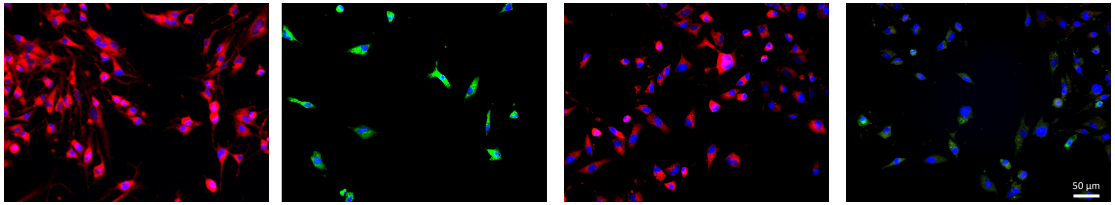

Supplement: Supplementary file 2 — Supporting File: advs74716‐sup‐0002‐Data.pdf [file ADVS-13-e18901-s002.pdf]
